# Effects of increasing respiratory rate on ventilatory efficiency and mechanical costs during low-tidal-volume ventilation: a prospective physiological pilot study

**DOI:** 10.1016/j.aicoj.2026.100098

**Published:** 2026-06-02

**Authors:** Carolin Jung, Erietta Markou, Kai Florian Storch, Jona Wassong, Hans-Joerg Gillmann, Thomas Stueber

**Affiliations:** Department of Anesthesiology and Intensive Care Medicine, Hannover Medical School, Hannover, Germany

**Keywords:** Mechanical ventilation, Respiratory rate, Dead space, Ventilatory efficiency, Respiratory physiology

## Abstract

**Background:**

During lung-protective ventilation, respiratory rate (RR) is frequently increased to compensate for hypercapnia. Despite its widespread use, however, the physiological consequences of this strategy remain incompletely understood. We aimed to characterize the physiological trade-offs of stepwise RR escalation at fixed tidal volume during controlled ventilation, with a particular focus on dead space partitioning, ventilatory efficiency, and energetic burden.

**Methods:**

In this prospective physiological crossover pilot study, 30 mechanically ventilated ICU patients underwent stepwise RR increases from 15 to 33 min^−1^ (primary analysis restricted to ≤27 min^−1^) at constant tidal volume (6 mL·kg^−1^ ideal body weight (IBW)) under two protocols: complete expiration and fixed I:E ratio (1:1.9). Dead space was assessed using the Enghoff modification of the Bohr equation at two predefined minute ventilation targets (100 and 150 mL·kg^−1^ IBW), with capnographic variables derived from quadratic mixed-effects models. Ventilatory efficiency was quantified using the ventilatory ratio (VR), the expected-to-observed PaCO_2_ response derived from the alveolar ventilation equation, and the incremental CO_2_ elimination per unit additional minute ventilation (ΔV̇CO_2_/ΔMV).

**Results:**

A 50% increase in minute ventilation (RR 17→25 min^−1^) reduced PaCO_2_ by 5.1 mmHg (95% CI 4.2–5.9), systematically less than predicted under the assumption of constant dead space fraction. Alveolar dead space increased from 132 to 189 mL (p < 0.001) and dead space fraction rose from 0.60 to 0.65. Incremental CO_2_ elimination efficiency declined with increasing RR (β = −0.95 ml CO_2_/L MV per min^−1^, 95% CI −1.41 to −0.49), suggesting plateau formation under complete expiration. Mechanical power increased exponentially, reaching +181% under complete expiration versus +122% under fixed I:E at 27 min^−1^. Baseline VR moderated V̇CO_2_ trajectories: patients with high VR tended to show declining CO_2_ elimination beyond 21–24 min^−1^, whereas low-VR-patients showed approximately linear increases.

**Conclusions:**

In this physiological study, RR escalation during low-tidal volume ventilation was associated with progressive decline in ventilatory efficiency, driven by increasing dead space fraction and compounded by rising CO_2_ rebreathing. These effects were most pronounced in patients with elevated baseline VR, supporting future investigation of individualized ventilation approaches that balance gas exchange efficiency against mechanical cost.

## Highlights

• Frequency-based minute ventilation augmentation encounters physiological limits.
• Increasing dead space and CO_2_ rebreathing diminish ventilatory efficiency.
• Mechanical power rises disproportionately while CO_2_ elimination gains diminish.
• Patients with higher ventilatory ratio derive the least benefit from higher rates.

## Graphical abstract

**Figure:**
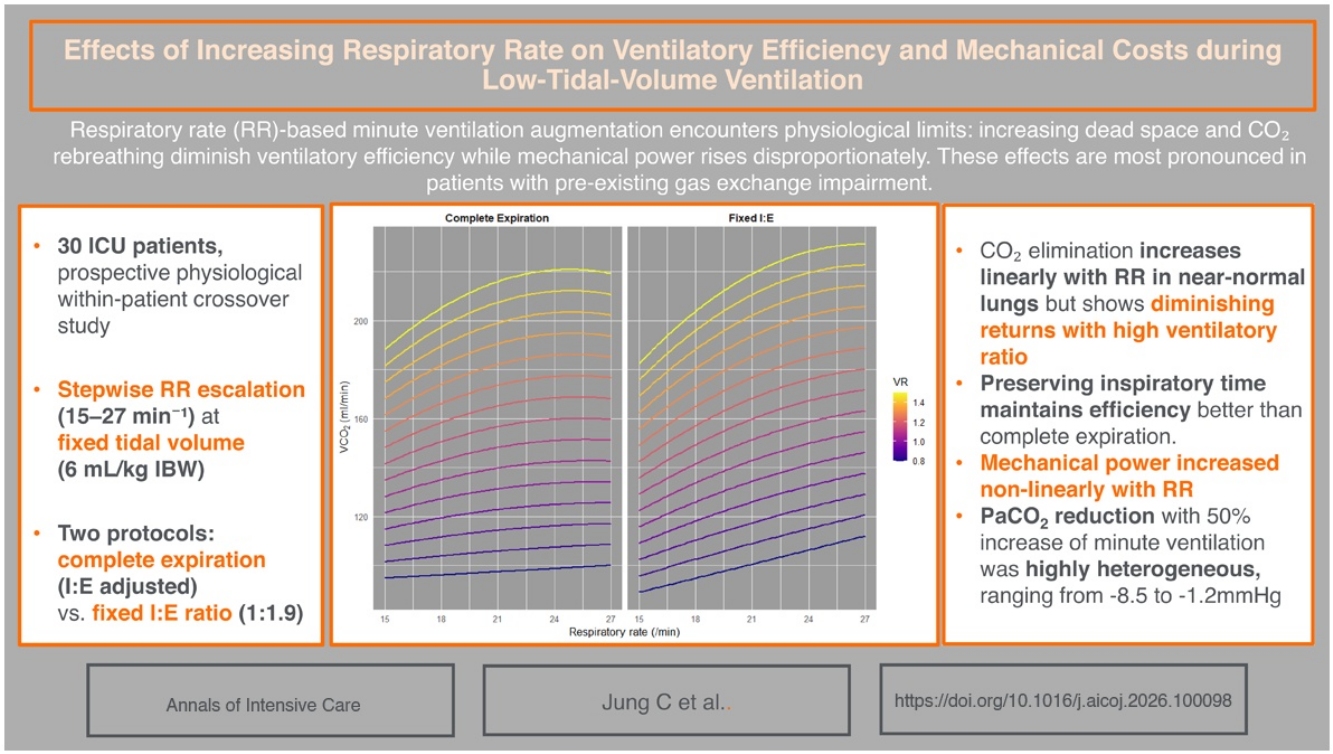

## 1 Introduction

In acute respiratory distress syndrome (ARDS), lung-protective ventilation with low tidal volumes is a cornerstone strategy to reduce ventilator-induced lung injury (VILI), but frequently induces hypercapnia [1]. While the importance of tidal volume in gas exchange and lung protection is well established, the physiological consequences of manipulating respiratory rate (RR) under lung-protective constraints have received far less systematic attention. In clinical practice, hypercapnia is commonly addressed by increasing RR, with frequencies ≥25 breaths per minute often applied during low-tidal ventilation [2]. Yet, experimental evidence indicates that the inspiratory flow profile, including inspiratory time, post-inspiratory pause, and flow waveform, influences carbon dioxide (CO_2_) clearance by modulating the mean distribution time available for gas mixing and diffusion [3,4]. These variables also influence the available expiratory time and may thereby affect air trapping. Consequently, increases in RR, which inherently alter cycle timing and inspiratory flow, do not necessarily translate into proportional improvements in CO_2_ elimination [5]. Together, these observations raise the question whether additional breaths deliver the expected gains in CO_2_ elimination or are increasingly lost to dead space ventilation. Elevated physiological dead space has been consistently associated with adverse outcomes in ARDS and other forms of acute respiratory failure [[6], [7], [8]], yet its dynamic response to changes in RR is largely unknown. Within the mechanical power framework, RR represents a major determinant of energy transfer to the respiratory system [9] and has been independently implicated in VILI across experimental models [[10], [11], [12]]. Whether the energetic cost of RR escalation is justified by corresponding gains in CO_2_ elimination, and how this trade-off varies across lung conditions, has not been systematically quantified in the clinical ICU setting. Volumetric capnography enables partitioning of dead space components [13,14] and real-time assessment of CO_2_ elimination efficiency [15]. We therefore conducted a prospective physiological study characterizing the trade-offs of stepwise RR escalation at fixed tidal volume in controlled ventilation. To address the uncertain role of air trapping in gas exchange efficiency during rate escalation, we compared two protocols: one avoiding air trapping entirely, the other allowing moderate trapping under clinically representative conditions. Integrating arterial blood gas analysis, volumetric capnography, and mechanical power decomposition in a within-patient crossover design, the study characterized the intra-individual physiological response to RR escalation.

## Material and methods

### Study design and setting

This prospective physiological within-patient crossover study was conducted in a mixed medical-surgical intensive care unit. Given the absence of prior data on stepwise RR escalation under controlled expiratory conditions, it was designed as a pilot investigation to provide initial effect-size estimates and protocol-feasibility data for a planned larger follow-up study. The study protocol was approved by the institutional ethics committee (No.11792-BO-S-2025), and written informed consent was obtained from all patients or their legal representatives prior to enrolment. This study was prospectively registered on May 7, 2025 in the German Clinical Trials Register (DRKS00036836), and recruitment was conducted between May and December 2025. The manuscript was prepared in accordance with the Strengthening the Reporting of Observational Studies in Epidemiology (STROBE) guidelines.

### Study objectives and pre-specified endpoints

This study examined RR effects on ventilatory efficiency in controlled mechanical ventilation. We pre-specified two hypotheses based on the conventional assumption that dead space remains stable across RR at fixed tidal volume: (1) alveolar dead space would remain constant during RR escalation (primary endpoint); (2) a 50% increase in minute ventilation would produce a proportional arterial CO_2_ reduction. These hypotheses served as testable a priori expectations against which observed physiological responses were evaluated. Beyond pre-specified endpoints, exploratory post-hoc analyses examined mechanical power, the simplified power index 4·ΔP + RR [16], incremental CO_2_ elimination efficiency, treatment response heterogeneity, and predictive value of baseline ventilatory ratio. These analyses were hypothesis-generating and should be interpreted with appropriate caution.

### Study population

We enrolled deeply sedated, passive adult patients requiring invasive mandatory mechanical ventilation, including patients following cardiac or major abdominal surgery and critically ill medical patients. Exclusion criteria were age below 18 years, pregnancy, elevated intracranial pressure, and extracorporeal membrane oxygenation. Patients had to be hemodynamically stable and received neuromuscular blockade (rocuronium) to ensure passivity. Ventilation was provided using Hamilton C6 ventilators (Hamilton Medical AG, Bonaduz, Switzerland) with mainstream volumetric capnography, except for two patients ventilated with the Hamilton T1. Positive end-expiratory pressure (PEEP) and fraction of inspired oxygen (FiO_2_) remained constant at levels determined by the treating clinician. Sedation was maintained with propofol or midazolam combined with sufentanil or hydromorphone.

### Measurements

RR effects were studied using two contrasting protocols (see Figure S1 for a detailed schematic of the study flow). Protocol A adjusted the inspiration-to-expiration (I:E) ratio at each step to ensure complete expiration (algorithm in Supplement), eliminating dynamic hyperinflation as a confounder. Protocol B applied a fixed I:E ratio (1:1.9), preserving proportional scaling of inspiratory and expiratory time across rate steps. Both protocols delivered volume-controlled ventilation with a 50% decelerating inspiratory flow pattern at a fixed tidal volume of 6 mL·kg^−1^ ideal body weight (IBW). This flow profile resembles pressure-controlled ventilation, the default mode in our setting, and produces lower peak inspiratory pressures (Ppeak) than a constant (square) flow pattern, particularly at higher RR.

We applied a sequential within-patient crossover design in which each patient served as their own control, allowing the effects of RR and cyclic timing, and associated variations in inspiratory flow, to be evaluated largely independent of between-patient variability. RR was increased stepwise by 3 min^−1^ from 15 to 33 min^−1^ under both protocols, in fixed order (Protocol A then B), with a 10-minute equilibration between protocols. Each RR step was maintained for a 2-minute equilibration before five consecutive breath cycles were averaged. Predefined safety criteria governed protocol discontinuation: EtCO_2_ decrease >30%, systolic blood pressure decrease >20%, plateau pressure (Pplat) >30 mbar, driving pressure (ΔP) >15 mbar, tidal volume >8 mL·kg^−1^ IBW, or Ppeak ≥40 mbar. Termination of Protocol A by a safety criterion did not preclude participation in Protocol B, provided baseline conditions had been restored. Within Protocol A, arterial blood gas (ABG) samples were obtained at two predefined minute ventilation targets of 100 and 150 mL·kg^−1^ IBW, corresponding to RR of 17 and 25 min^−1^, respectively, given the fixed tidal volume. Each measurement was preceded by a 5-minute equilibration with unchanged ventilator settings.

### Variables

Measured variables are depicted in Table S2, and included partial pressure of end-tidal CO_2_ (PetCO_2_), expiratory and inspiratory volume of CO_2_ per breath (VeCO_2_, ViCO_2_), and volume of CO_2_ eliminated per minute (V̇CO_2_). Static respiratory mechanics were assessed using inspiratory and expiratory hold maneuvers lasting 3–4 seconds to ensure equilibration. Mechanical power (MP) was calculated as MP = 0.1 × RR × VTi × (Ppeak – 0.5 × ΔP) [9] and decomposed into elastic, resistive, and static components (full equation in Supplement).

### Dead space partitioning

Physiological dead space (VDphys) was calculated using the Enghoff modification [17]: VDphys = VTe × (PaCO_2_ − P̄ECO_2_) / PaCO_2_, where P̄ECO_2_ = (VeCO_2_ / VTe) × 713 mmHg. Unlike the original Bohr equation, the Enghoff modification substitutes arterial PaCO_2_ and thus additionally captures intrapulmonary shunt and ventilation–perfusion mismatch. Airway dead space (VDaw) [18,19] was measured using the pre-interface expirate (PIE) method [20] integrated in the Hamilton C6 volumetric capnography module. Alveolar dead space was estimated as VDphys − VDaw, noting that this Enghoff-derived measure represents a composite of true alveolar dead space and ventilation–perfusion inefficiency. VDphys was determined at the two predefined minute ventilation targets, whereas VDaw was available at all RR levels. Because capnographic measurements (RR 15, 18, 21, 24, 27 min^−1^) did not coincide with ABG sampling points (RR 17 and 25 min^−1^), patient-specific capnographic values were derived from quadratic mixed-effects models fitted to complete expiration data.

### Ventilatory efficiency

V̇CO_2_ was derived from capnographic data after a 2-minute equilibration at each RR step, an interval within which V̇CO_2_ has been validated as a measure of effective alveolar ventilation [15]. The ventilatory ratio (VR) was calculated at both minute ventilation targets as VR = (MV × PaCO_2_) / (IBW × 100 × 37.5), where MV is measured minute ventilation (mL·min^−1^), PaCO_2_ is arterial carbon dioxide tension (mmHg), IBW is ideal body weight (kg), and 37.5 mmHg represents the expected PaCO_2_ in normal lungs ventilated at the predicted minute ventilation [21]. To quantify efficiency loss with RR escalation, the observed PaCO_2_ change between the two MV targets was compared with the expected change from the alveolar ventilation equation. Assuming constant dead space fraction and metabolic CO_2_ production, PaCO_2_ is inversely proportional to MV: PaCO_2expected_ = PaCO_2 MV100_ × (MV_100_ / MV_150_), where MV_100_ and MV_150_ are measured minute ventilations at the two sampling points. Incremental CO_2_ elimination efficiency (ΔV̇CO_2_/ΔMV) was calculated between consecutive RR steps. The relationship between baseline VR and treatment response was assessed using Spearman rank correlation.

### Statistical analysis

Mixed-effects models were fitted for all outcomes with RR (centered at 15 min^−1^), ventilation protocol, and their interactions as fixed effects, and patient-specific random intercepts and slopes. Linear, quadratic, and exponential (log-transformed) specifications were compared by the Akaike Information Criterion (AIC), with the Bayesian Information Criterion (BIC) reported for sensitivity (Table S6). Estimated marginal means were derived at each RR level using Satterthwaite-adjusted degrees of freedom. Incremental efficiency was analysed using linear mixed-effects models with patient-specific random intercepts. The primary endpoint was tested at α = 0.05 without adjustment; exploratory outcomes were corrected using the Benjamini–Hochberg procedure (q < 0.05) within physiologically related families. Protocol discontinuation due to predefined safety criteria produced missing-not-at-random patterns (Figure S2). The primary analysis was therefore restricted to RR ≤ 27 min^−1^, where patient retention exceeded 80% in both protocols. Given the pilot design, sample size was determined by feasibility rather than formal power analysis. Detailed modelling procedures and ventilator circuit configuration are provided in the Supplement. Analyses were performed using R (version 4.5.1) with lme4, lmerTest, emmeans, and performance packages.

## Results

### Patient characteristics

Thirty patients completed the protocol (29 with paired ABG data, 30 with full ventilator data). Patient characteristics are shown in Table 1. No adverse events, respiratory deterioration, or hemodynamic compromise occurred. All discontinuations followed predefined safety criteria (Table S1), with protocols differing in the proportion reaching the highest rates (Figure S3). During the equilibration interval between both protocols, PetCO_2_ returned to within 15% of baseline in 97% of patients (29/30), with no significant difference between protocols (42.0 ± 8.2 vs. 41.2 ± 10.0 mmHg; p = 0.163), suggesting adequate washout. Protocol A maintained 100% retention through 24 min^−1^ before declining steeply to 17% at 33 min^−1^. Protocol B showed earlier but more gradual attrition, retaining 53% of patients at 33 min^−1^.

**Table 1:** Baseline characteristics.

Parameters	All patients (n = 30)	Active lung condition (n = 21)	No active lung condition (n = 9)	SMD
Age	62 ± 11	60 ± 12	68 ± 8	0.833
Sex, male	21 (70%)	14 (67%)	7 (78%)	0.25
Height, cm	175.9 ± 11.0	176.0 ± 11.7	175.9 ± 9.7	0.006
Weight, kg	94.7 ± 31.6	101.0 ± 34.9	80.0 ± 14.5	0.785
IBW, kg	69.8 ± 11.5	69.7 ± 12.2	70.1 ± 10.2	0.035
BMI	30 ± 9	32 ± 10	26 ± 4	0.839
CCI	4 (3–8)	4 (4–8)	4 (3–5)	0.27
PaO_2_/FiO_2_ ratio	286.1 ± 89.6	260.3 ± 80.9	346.1 ± 83.4	1.044
PetCO_2,_ mmHg	38.9 ± 6.5	40.4 ± 7.2	35.7 ± 2.3	0.873
Primary reason for ICU admission:				
A) Major surgery				
- abdominal	5 (17%)	3 (14%)	2 (22%)	0.207
- cardiac	9 (30%)	2 (10%)	7 (78%)	1.897
B) Medical				
- ARDS	4 (13.5%)	4 (19%)		0.686
- Sepsis	4 (13.5%)	4 (19%)		0.686
- Uterus rupture	1 (3%)	1 (5%)		0.316
C) Neurologic				
- Ischemic stroke	2 (7%)	2 (10%)		0.459
- Intracranial bleeding	3 (10%)	3 (14 %)		0.577
- Hypoxic-ischemic Encephalopathy	1 (3%)	1 (5%)		0.316
- Guillain-Barre syndrome	1 (3%)	1 (5%)		0.316
Active lung condition:				
- ARDS	8 (27%)	8 (38%)	0 (0%)	1.109
- Unilateral Pneumonia	5 (16.7%)	5 (24%)	0 (0%)	0.791
- Obesity-related respiratory impairment	7 (23.3%)	7 (33%)	0 (0%)	1
- Obstructive lung disease	10 (33%)	10 (48%)	0 (0%)	1.348
Systemic inflammation:				
- White blood cell count, ×10⁹/L	11.4 (7.2–16.9)	11.9 (9.6–17.0)	8.2 (6.0–14.1)	0.555
- C-reactive protein, mg/L	125.1 ± 104.2	155.7 ± 110.2	53.8 ± 28.3	1.267
Baseline Respiratory mechanics:				
- Cstat, mL/mbar	62.9 ± 25.3	56.4 ± 19.1	77.2 ± 32.2	0.786
- Rinsp, mbar/L/s	6.1 ± 3.5	6.2 ± 3.3	5.9 ± 4.0	0.082
- RCexp, s	0.7 ± 0.2	0.7 ± 0.1	0.7 ± 0.2	0.222
Baseline clinical ventilator settings before study protocol:				
- Set PEEP, mbar	8 (5–10)	10 (5–10)	5 (5−5)	1.531
- Set FiO2	0.4 (0.3−0.4)	0.4 (0.3−0.4)	0.3 (0.3−0.4)	0.173
- Set Respiratory Rate	14 (12–16)	15 (12–18)	12 (12−12)	1.296
- Pplat,mbar	17 ± 5	19 ± 5	13 ± 2	1.651
- Ppeak, mbar	19 ± 5	21 ± 5	15 ± 3	1.651
- ΔP, mbar	9.2 ± 2.5	10 ± 3	8 ± 1	1.169
- Vt/kg IBW	7.4 (6.7–8.1)	7.2 (6.9–7.2)	7.7 (6.64–8.4)	0.535
In-hospital mortality	4 (13%)	4 (19%)	0 (0%)	0.686

Data are presented as mean ± SD for normally distributed continuous variables, median (25th–75th percentile) for non-normally distributed continuous variables, and n (%) for categorical variables. Patients were stratified by lung health status into those without active lung condition (postoperative patients with healthy lungs) and those with active lung condition (ARDS, pneumonia, obesity-related respiratory impairment, or obstructive pulmonary disease). Diagnoses under ‘Active lung condition’ are not mutually exclusive. ‘Primary reason for ICU admission’ reflects admission category and may differ from pulmonary diagnoses present at the time of study measurements. Standardized Mean Difference (SMD) was used to assess group differences, where values of 0.2, 0.5, and 0.8 are considered small, moderate, and large, respectively. Abbreviations: ARDS, acute respiratory distress syndrome; BMI, body mass index; CCI, Charlson Comorbidity Index; CRP, C-reactive protein; Cstat, static compliance; ΔP, driving pressure; FiO_2_, fraction of inspired oxygen; IBW, ideal body weight; I:E, inspiratory-to-expiratory ratio; MV, minute ventilation; PaO_2_/FiO_2_, ratio of arterial oxygen partial pressure to fractional inspired oxygen; PEEP, positive end-expiratory pressure; PetCO_2_, end-tidal carbon dioxide; Pplat, plateau pressure; Ppeak, peak inspiratory pressure; RCexp, expiratory time constant; RR, respiratory rate; Vt, tidal volume; WBC, white blood cell count.

### Dead space response to RR escalation

Following a 50% increase in minute ventilation through RR escalation (from 17 to 25 min^−1^ at constant tidal volume), estimated alveolar dead space increased from 132.0 to 188.5 mL (mean difference +56.9 mL, 95% CI 48.9–64.9). VDphys rose from 245.7–274.2 mL (+27.7, 95% CI 21.3–34.1), while VDaw decreased from 114.7 to 85.7 mL (−29.1, 95% CI −32.0 to −26.1). Dead space fraction rose from 0.60 to 0.65 (+0.045, 95% CI 0.033 to 0.057). As RR increased, VDaw progressively declined under complete expiration (123 to 104 mL, −15%) but remained largely stable under fixed I:E (123 to 121 mL, −2%). The interaction between RR and ventilation protocol was statistically significant (p < 0.001; Tables S4 and S5).

### Arterial blood gas response

**Fig. 1:**
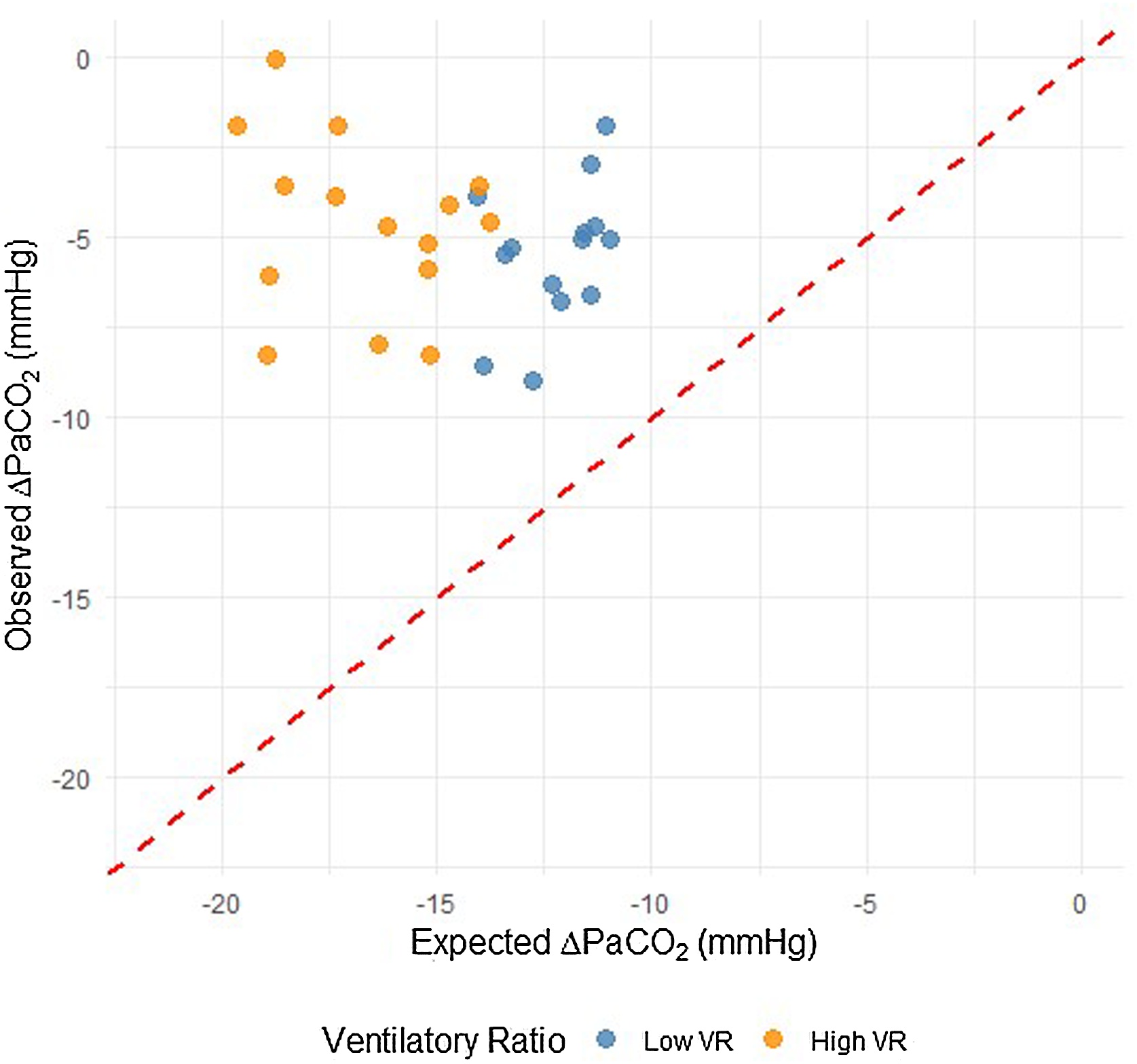
Expected versus observed PaCO_2_ response to respiratory rate-driven minute ventilation augmentation. Each point represents one patient (n = 29). The x-axis shows the expected absolute change in PaCO_2_ assuming constant dead space fraction, derived from the alveolar ventilation equation as (MV_pre/MV_post − 1) × PaCO_2__baseline, where MV_pre and MV_post are the measured minute ventilations at the two sampling points (median ratio 0.667). The y-axis shows the observed PaCO_2_ change. The dashed red identity line indicates perfect agreement; points above this line reflect less PaCO_2_ reduction than predicted. Patients were stratified by median baseline ventilatory ratio (VR) into low VR (blue) and high VR (orange) groups. Higher baseline VR was associated with greater deviation from the expected response (Spearman's ρ = 0.78, p < 0.001).

Increasing minute ventilation from 100 to 150 mL·kg^−1^·min^−1^ (IBW) reduced PaCO_2_ from 43.4 to 38.2 mmHg (−5.1, 95% CI −5.9 to −4.2) and increased pH from 7.38 to 7.43 (+0.05, 95% CI 0.04 to 0.06; Figure S9). The observed PaCO_2_ reduction was systematically smaller than predicted by the alveolar ventilation equation assuming constant dead space fraction, with greater deviation in patients with higher baseline VR (ρ = 0.78, 95% CI 0.57 to 0.90; Fig. 1). VR rose from 1.15 to 1.53 (+0.38, 95% CI 0.32 to 0.44). PaO_2_/FiO_2_ was unchanged (+6.5, 95% CI −6.6–19.6).

Beyond the prespecified endpoints, exploratory analyses examined CO_2_ elimination efficiency, mechanical power, and treatment response heterogeneity.

### CO_2_ elimination and ventilatory efficiency

**Fig. 2:**
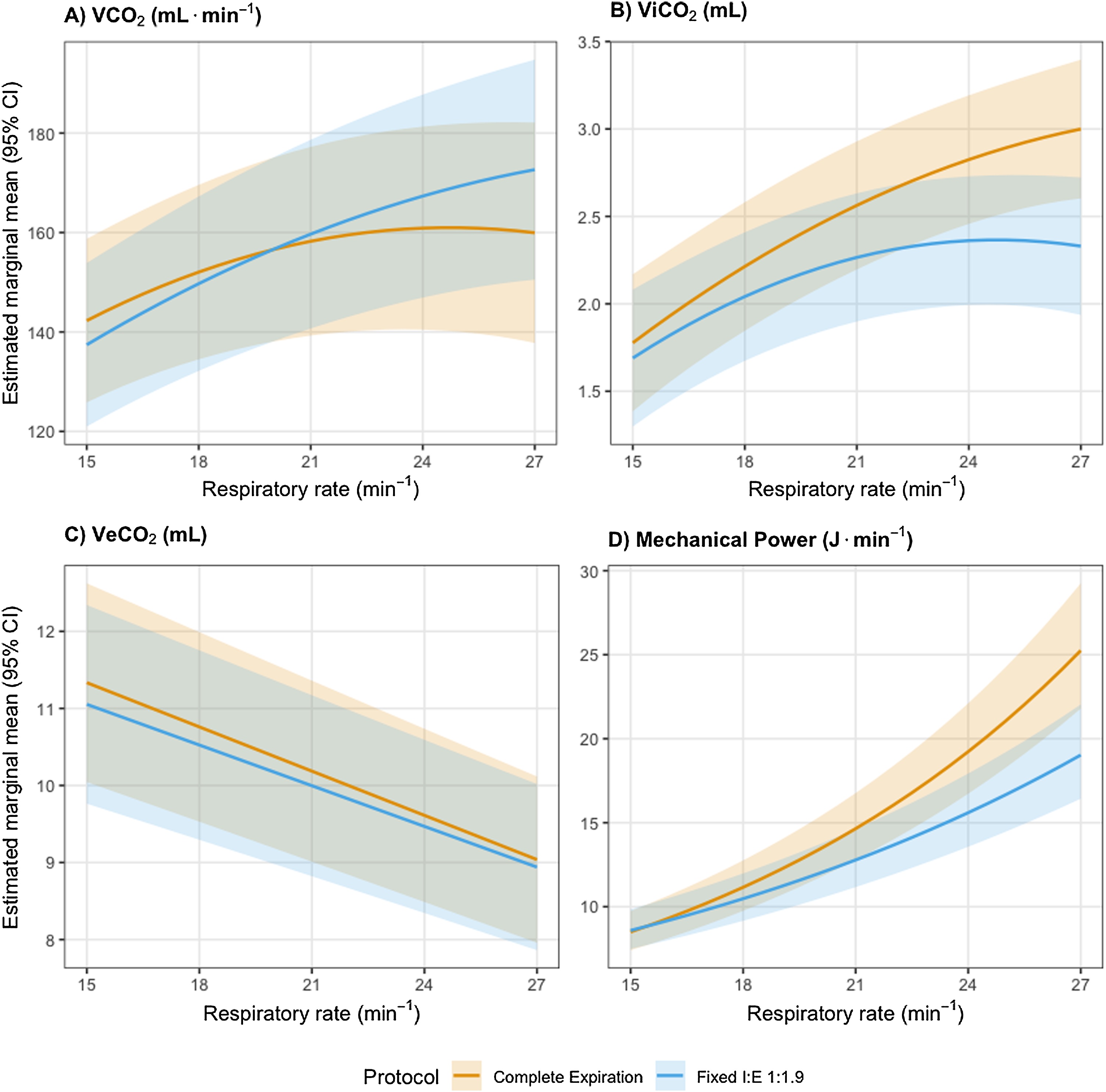
CO_2_ elimination parameters and mechanical power across respiratory rate steps. Estimated marginal means with 95% confidence intervals from mixed-effects models with outcome-specific functional forms (A and B: quadratic; C: linear; D: exponential) under complete expiration (orange) and fixed I:E 1:1.9 (blue) protocols. A) Net CO_2_ elimination (V̇CO_2_) increased modestly at lower rates, with less deceleration under fixed I:E than under complete expiration, where gains tended to level off. B) Re-inspired CO_2_ volume per breath (ViCO_2_) increased in both protocols, more markedly under complete expiration. C) Expired CO_2_ volume per breath (VeCO_2_) declined progressively in both protocols. D) Total mechanical power increased exponentially, with pronounced protocol-specific divergence at higher respiratory rates. Analysis restricted to respiratory rates 15–27 min^−1^ (n = 30 patients, 279–289 observations per outcome).

Despite an 80% increase in minute ventilation across the RR range, V̇CO_2_ increased only modestly, which may reflect declining VeCO_2_ per breath and increasing re-inspired CO_2_ (ViCO_2_: +60% complete expiration, +39% fixed I:E). Under complete expiration, V̇CO_2_ appeared to approach a plateau (peak 160 mL·min^−1^ at RR 24 min^−1^), whereas under fixed I:E, increases continued but appeared to slow at higher rates (138–172 mL·min^−1^; detailed trajectories in Fig. 2 and Table S4).

**Fig. 3:**
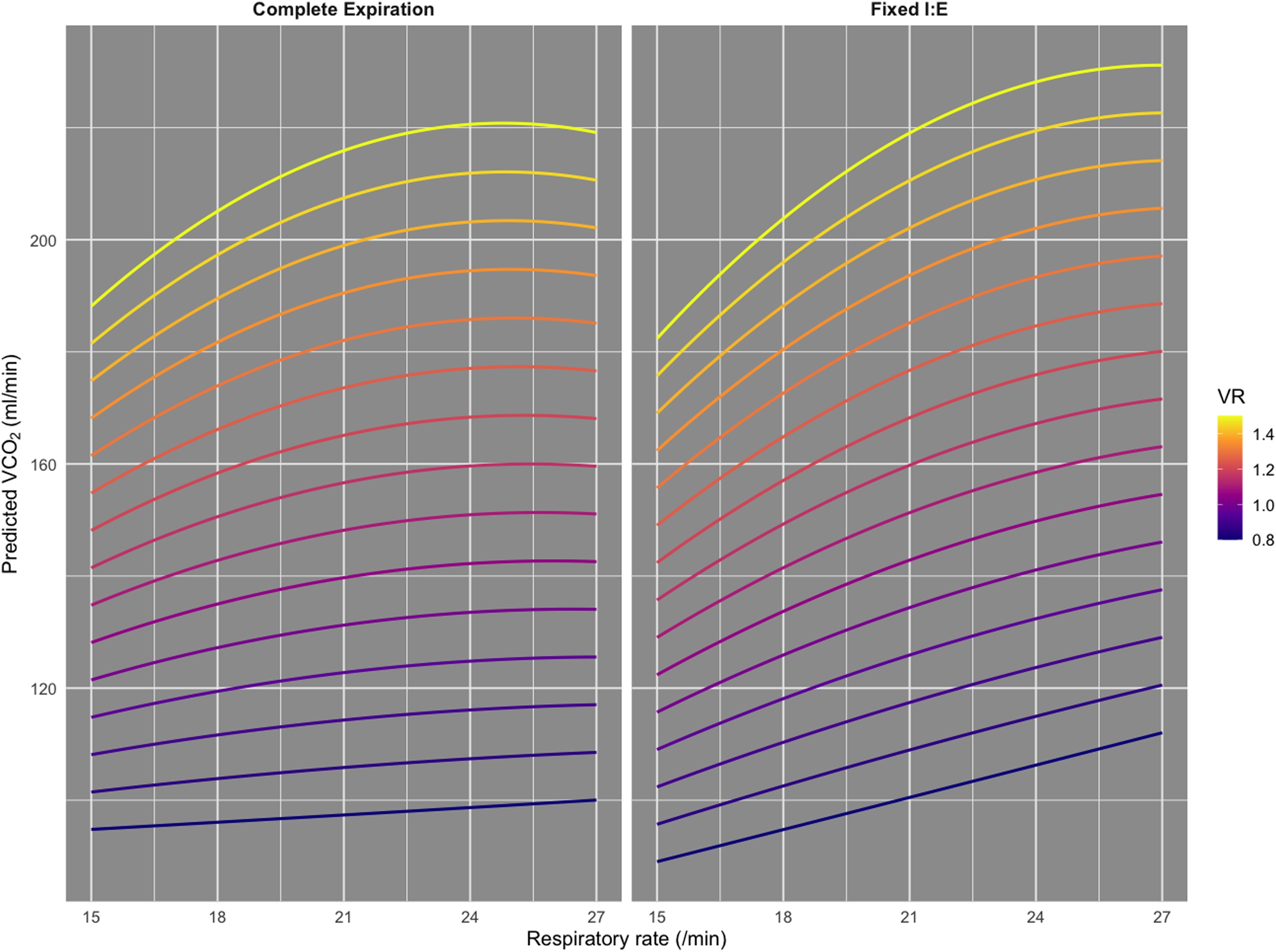
Ventilatory ratio moderates the relationship between respiratory rate and CO_2_ elimination. Model-predicted V̇CO_2_ trajectories as a function of respiratory rate under complete expiration (left, variable inspiratory time ensuring full exhalation) and fixed I:E (right, ratio 1:1.9). Each line represents the predicted V̇CO_2_ for a given baseline ventilatory ratio (VR), ranging from 0.80 (purple) to 1.50 (yellow) in 0.05 increments (color scale: dark = low VR, light = high VR). Predictions are derived from a quadratic mixed-effects model incorporating VR as a continuous, centered moderator of both linear and quadratic respiratory rate effects (n = 29 patients, 279 observations). The vertical spread reflects the main effect of baseline VR on V̇CO_2_. Analysis restricted to respiratory rates 15–27 min^−1^. Model coefficients are reported in the text. Predictions suggest that under complete expiration, patients with high VR (≥1.2) may follow an inverted-U pattern with model-predicted V̇CO_2_ declining at respiratory rates above 21–24 min^−1^, whereas patients with low VR appear to show an approximately linear increase. Under fixed I:E, predicted V̇CO_2_ trajectories tended to be steeper across all VR levels, with flattening at high VR but no frank decline in the observed range. These patterns reflect model-based extrapolations and should be interpreted cautiously given the sample size.

V̇CO_2_ trajectories were significantly moderated by baseline VR (Fig. 3; observed individual trajectories shown in Figure S8). Both slope and curvature of the V̇CO_2_-RR relationship varied with baseline VR (RR × VR interaction β = 3.07, p = 0.039; RR² × VR interaction β = −0.49, p = 0.012), although the magnitude and shape of these effects should be interpreted cautiously given sample size and variability. Under complete expiration, high-VR patients exhibited trajectories consistent with an inverted-U pattern with V̇CO_2_ declining beyond 21–24 min^−1^, whereas low-VR patients showed continued increases across the studied range. Fixed I:E produced steeper V̇CO_2_ increases across all VR levels (protocol × RR interaction β = 1.48, p < 0.001), though gains appeared to decelerate at higher VR.

Incremental CO_2_ elimination efficiency (ΔV̇CO_2_/ΔMV) declined with increasing RR (β = −0.95 mL CO_2_/L MV per min^−1^, 95% CI −1.41 to −0.49; Table S3). Under complete expiration, confidence intervals included zero from the third rate step onward (21→24 min^−1^), indicating substantial uncertainty in the direction of the effect at higher rates, whereas fixed I:E maintained positive incremental elimination across all steps.

### Mechanical power and energetic cost

**Fig. 4:**
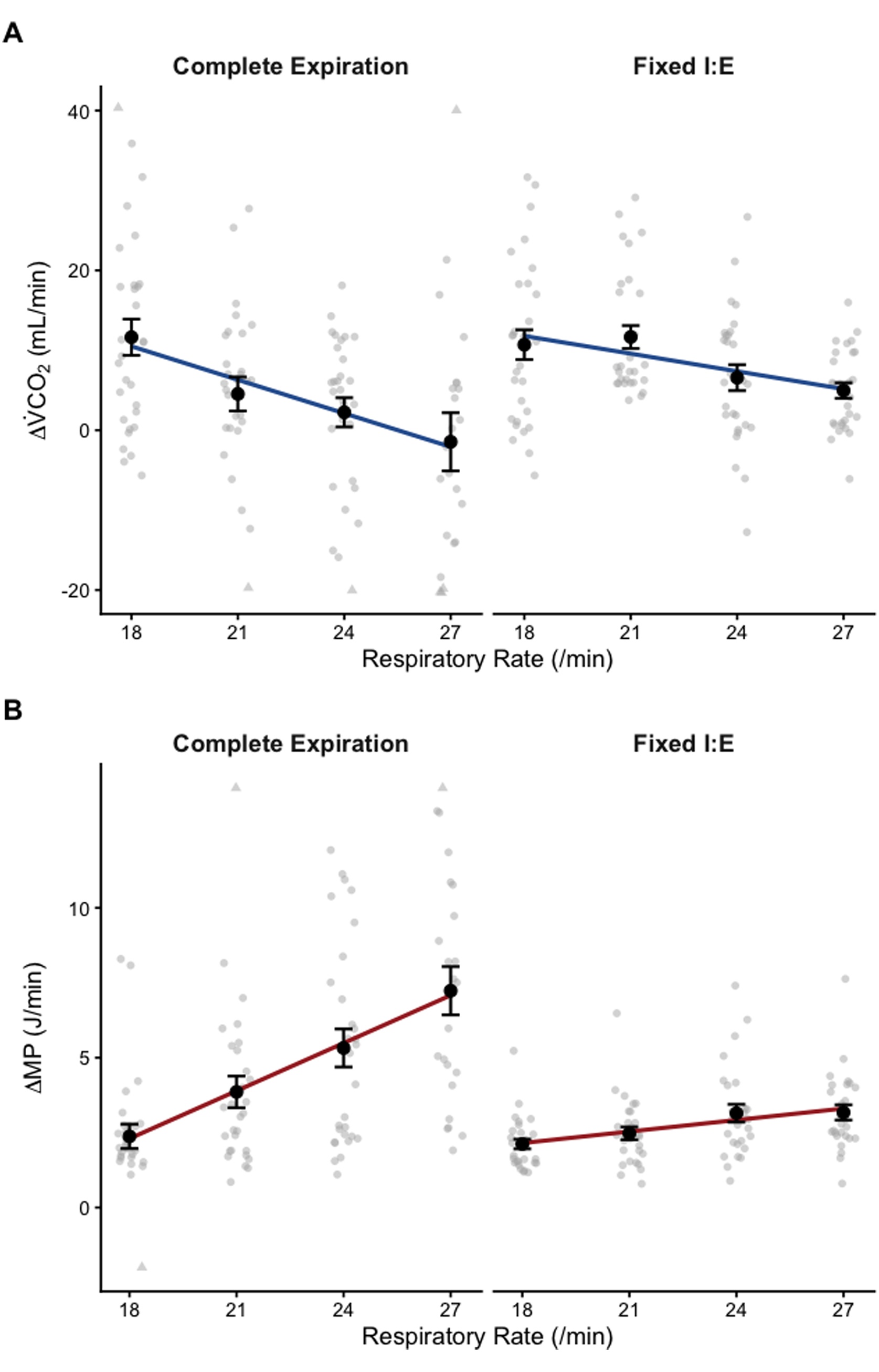
Marginal changes in CO_2_ elimination and mechanical power across respiratory rate increments under both protocols. (A) Change in CO_2_ elimination (ΔV̇CO_2_) and (B) change in mechanical power (ΔMP) between consecutive respiratory rate steps under the Complete Expiration protocol (Protocol A) and the Fixed I:E protocol (Protocol B). Gray points represent individual observations; values exceeding the displayed y-axis range (ΔV̇CO_2_ > 30 or < −20 mL·min^−1^; ΔMP > 12 or < −2 J·min^−1^) are shown as triangles at the corresponding axis limit. Black points with error bars indicate group means ± standard error of the mean (SEM). Colored lines represent linear mixed model fits with random intercepts for individual patients. ΔV̇CO_2_ decreased significantly with increasing respiratory rate under both protocols (Protocol A: β = −1.40 mL·min^−1^ per step, p < 0.001; Protocol B: β = −0.76 mL·min^−1^ per step, p < 0.001), with no statistically significant difference in slopes between protocols (interaction p = 0.14). ΔMP increased significantly under both protocols (Protocol A: β = +0.56 J·min^−1^ per step, p < 0.001; Protocol B: β = +0.12 J·min^−1^ per step, p < 0.001), with a markedly steeper increase under Protocol A (interaction p < 0.001). Together, these findings indicate diminishing returns in CO_2_ elimination at progressively higher mechanical costs, with a substantially more pronounced trade-off under the Complete Expiration protocol.

Total MP increased exponentially with RR, with protocol-specific divergence (Fig. 2). At baseline, both protocols delivered comparable power (9.3 vs. 9.1 J·min^−1^). Complete expiration reached 26.1 J·min^−1^ at RR 27 min^−1^ (+181%), versus 20.2 J·min^−1^ under fixed I:E (+122%), driven primarily by increased resistive work at higher inspiratory flows (Figure S4). Incremental MP rose progressively with each rate step (β = +0.11 J·min^−1^ per step, 95% CI 0.08 to 0.15), contrasting with the declining incremental CO_2_ elimination described above (Fig. 4, Table S7). 4·ΔP + RR rose approximately linearly with only slight deceleration at higher RR, and without protocol-specific rate interaction (Table S5, Figure S5).

### Treatment response heterogeneity

Individual PaCO_2_ responses ranged from −8.5 to −1.2 mmHg (Figure S12). Patients without active lung condition showed larger PaCO_2_ reductions (−6.1 ± 1.4 vs. − 4.7 ± 2.3 mmHg; mean difference 1.4, 95% CI 0.0–2.9) and pH increases (+0.069 ± 0.014 vs. + 0.042 ± 0.018; mean difference 0.027, 95% CI 0.014 to 0.041). PaO_2_/FiO_2_ responses diverged markedly (near-normal lungs: +33.6 ± 24.8 vs. active lung condition: −4.4 ± 31.0; mean difference 38.0, 95% CI 14.3–61.7). Across the cohort, baseline VR predicted VR worsening during MV augmentation (r = 0.82, 95% CI 0.65 to 0.91), with larger increases at high baseline VR (+0.48 ± 0.14 vs. + 0.27 ± 0.07; mean difference 0.21, 95% CI 0.13 to 0.30).

Higher baseline VR was associated with a 2.6-fold increase in MP per unit VR across all RR levels (95% CI 1.64–4.23; β = 0.97, p < 0.001, Figure S6). The proportional MP increase per RR step (∼10%) was comparable across VR levels (interaction β = −0.01, p = 0.57). 4·ΔP + RR showed the same qualitative pattern (Figure S7).

Sensitivity analyses (Figures S11, Table S9), signal stability (Table S10), and detailed respiratory parameter trajectories are reported in the Supplement.

## Discussion

Our findings suggest that increasing RR from 15 to 27 breaths per minute at constant tidal volume in ICU patients receiving controlled ventilation yields only modest arterial CO_2_ reduction despite substantially increased minute ventilation, indicating that a large fraction of additional ventilation did not contribute to effective alveolar CO_2_ elimination. This limited benefit was accompanied by rising VDphys, declining ventilatory efficiency, and a disproportionate increase in MP. Baseline VR moderated the response: V̇CO_2_ elimination appeared to reach a plateau that tended to emerge earlier and more pronounced in patients with higher VR, particularly under shortened inspiratory times.

Two ventilatory timing strategies with distinct physiological trade-offs were examined. Complete expiration prevented dynamic hyperinflation but required progressively higher inspiratory flows due to shortened inspiratory time, increasing resistive work and substantially raising MP. Re-inspired CO_2_ volume also increased with RR, attenuating net CO_2_ elimination per minute. Although small per breath, its relative contribution increased with RR, consistent with reports of amplified CO_2_ re-inspiration under high flow and short inspiratory time [3,22,23]. In contrast, a fixed I:E ratio produced steeper V̇CO_2_ increases across VR levels, with less deceleration at higher rates. Lower inspiratory flows reduced CO_2_ re-inspiration, while preserved inspiratory time likely sustained mean distribution time relevant for intra-alveolar gas distribution and diffusion, both effects gaining relevance at higher RR [3]. This came at the cost of modest intrinsic PEEP, which, unlike earlier reports [4], did not raise Pplat or ΔP within the studied range. Preserved ΔP across both strategies suggests that dynamic strain remained largely unchanged despite marked differences in inspiratory flow and Ppeak.

Contrary to the hypothesis, estimated alveolar dead space increased during RR escalation despite complete expiration. The observed PaCO_2_ reduction was systematically smaller than predicted by the alveolar ventilation equation assuming constant dead space fraction, with a concurrent rise in Enghoff dead space fraction. Baseline VR strongly predicted the PaCO_2_ response to increased minute ventilation, but did not correlate with the magnitude of dead space expansion. VDaw decreased under complete expiration but remained stable under fixed I:E. With external factors (tidal volume, apparatus configuration, PEEP) constant, this reduction likely reflects a flow- and time-dependent phenomenon rather than anatomical changes. However, it was offset by a rising VDphys, suggesting no net gain in gas exchange efficiency. This dissociation aligns with reports of increasing ventilation heterogeneity and pendelluft at high inspiratory flows [24], potentially reflecting inadequate filling of lung units with longer time constants during shortened inspiration.

Instrumental dead space deserves particular consideration during RR escalation at low tidal volumes, as the dead space gas is rebreathed more frequently, amplifying losses in effective alveolar ventilation [2]. In our protocol, all patients were ventilated with a heat-and-moisture exchanger (HME) and a minimized circuit, yielding 41.3 mL instrumental dead space distal to the Y-piece. In routine practice, however, it is often substantially higher: combining a HME (30–100 mL), a catheter mount (10–60 mL), and additional connectors for capnography, flow measurement, aerosol delivery, or closed suction can reach 200 mL [25], plausibly aggravating efficiency loss at higher rates. At high minute ventilation, instrumental dead space should be minimized using heated humidification (rather than HMEs) and minimal circuit configuration.

Total MP increased exponentially with RR, with protocol-specific divergence driven predominantly by resistive work at the higher inspiratory flows required under complete expiration. While elevated MP has been linked to VILI [9,26], validated thresholds and prospective evidence supporting MP as a therapeutic target are lacking. The framework’s strength lies in incorporating dynamic components, namely RR and flow-dependent resistive work, that static pressure parameters fail to capture. Its clinical interpretation, however, requires caution, as MP aggregates distinct physical forces without differentiating their respective injurious potential. Whereas ΔP is widely accepted as a marker of harmful elastic strain, the contribution of resistive and PEEP-derived energy remains debated [16,27]. Although experimental data suggest that high inspiratory flows may exert injurious effects [24,28], the magnitude of this contribution is uncertain. MP also disregards regional lung heterogeneity and local stress risers, which may trigger injury even at low global power. Since the rise in MP was driven by the resistive component while ΔP remained largely unchanged, the reported increase may overstate the actual injurious load. As a complementary metric, the simplified index 4·ΔP + RR increased more modestly than total MP, though still approximately linearly. While neither metric captures ventilatory heterogeneity, the simplified index omits resistive and PEEP-derived energy, confining it to variables with established injurious potential and providing a more conservative estimate of the mechanical burden.

Whether lower RR strategies confer clinical benefit also remains uncertain, as consistent effects on biological markers of lung injury or clinical outcomes have not been demonstrated [29]. Experimental data suggest that the injurious potential of RR may depend on concurrent conditions, including tidal volume, inspiratory flow [30], and susceptibility to overdistension [11]. Patients most likely to receive high RR strategies are those with the greatest baseline gas exchange impairment, in whom frequency-based CO_2_ elimination is least efficient and most energetically costly. This therapeutic dilemma contributes to the discussion that, under lung-protective constraints, moderate RR with modestly higher tidal volumes may offer a more favorable balance between gas exchange and energetic burden than aggressive frequency escalation [31,32]. This interpretation is strictly hypothesis-generating and should not be viewed as a clinical recommendation.

An important methodological consideration is the distinction between steady-state requirements for different outcome variables. Capnographic parameters were measured after a 2-minute equilibration at each RR step. V̇CO_2_ reflects changes in effective alveolar ventilation and accurately predicts the subsequent PaCO_2_ change within this timeframe after a ventilator step change in quasi-stable conditions. This proportionality is transient, as V̇CO_2_ subsequently returns toward the metabolic baseline once blood and tissue CO_2_ stores re-equilibrate. PaCO_2_ equilibration requires substantially longer [15] and depends on the magnitude of ΔRR, with shorter times after rate increases than decreases [33]. Assuming t_eq ≈ 0.5 × |ΔRR|, the largest cumulative change between consecutive blood gases (8 min^−1^) corresponds to an expected equilibration time of approximately 4 min. The median ABG interval was 20 (IQR 18–24) minutes, with 5 min of unchanged ventilator settings before each sample. While incomplete PaCO_2_ equilibration cannot be fully excluded, particularly with elevated dead space, the core finding of diminishing CO_2_ elimination efficiency does not rest on PaCO_2_ alone. The diminishing V̇CO_2_ gains at higher rates were derived from capnographic data using a validated method for real-time monitoring of effective alveolar ventilation [15] and are not subject to these equilibration constraints.

Several limitations must temper any clinical inference. First, measurements were performed under deep sedation with neuromuscular blockade, and responses may differ in patients with preserved respiratory drive or patient–ventilator interaction. Second, the fixed protocol sequence may have introduced carryover effects, although PetCO_2_ normalization before the second sequence suggests adequate washout. Third, the single-center design, modest sample size, and non-random attrition at frequencies ≥30 min^−1^ limit generalizability to specific subgroups or extreme rates. Fourth, PaCO_2_ responses should be interpreted as near rather than definitive steady-state effects, and were assessed only under complete-expiration, leaving uncertain whether similar patterns apply to fixed I:E. Fifth, absolute PaCO_2_ depends on metabolic CO_2_ production. Despite constant sedation, body temperature, and hemodynamics, residual heterogeneity in metabolic rate likely contributed to between-patient variability. Sixth, we used VCV with a decelerating flow, whereas square flow is more common in some settings. Ppeak and derived MP are not directly transferable between waveforms, as decelerating flow typically yields lower Ppeak than constant flow at equivalent tidal volumes. Dead space indices may also differ modestly through effects on mean distribution time and end-inspiratory pressure, although the evidence is conflicting [[34], [35], [36]]. Seventh, outcome variance was dominated by between-subject heterogeneity (body size, metabolism, lung mechanics), reflected in a low marginal but high conditional R². Against this baseline, a 3 breaths·min^−1^ change is mathematically expected to contribute modestly to total variance. Although the mixed-effects framework isolates a consistent intra-individual response, the small fixed effects caution against population-level extrapolation, particularly given the moderation of the V̇CO_2_ response by baseline VR. Finally, the physiological nature of the endpoints precludes conclusions regarding VILI or clinical outcomes, and all findings are hypothesis-generating.

## Conclusion

In this physiological crossover pilot study, RR escalation from 15 to 27 breaths min^−1^ at constant tidal volume produced modest arterial CO_2_ reductions but substantial increases in physiological dead space and mechanical power, with clear deterioration in ventilatory efficiency at higher rates. Patients with baseline gas exchange impairment derived the least benefit while incurring the greatest energetic penalty. These findings highlight the need for individualized ventilatory strategies balancing gas exchange efficiency against mechanical power under lung-protective constraints.

## Authors' contributions

CJ: Conceptualization, Methodology, Formal analysis, Investigation, Data curation, Writing – original draft, Project administration. TS: Conceptualization, Methodology, Formal analysis, Investigation, Data curation, Writing – original draft. HJG: Conceptualization, Methodology, Writing – review and editing. JW: Investigation, Writing – review and editing. EM: Investigation, Writing – review and editing. KS: Investigation, Writing – review and editing. All authors have read and approved the final manuscript.

## Consent for publication

Not applicable.

## Ethics approval and consent to participate

The Hannover Medical School Ethics Committee, Hannover, Germany (Chairperson Prof. B Schmidt), approved this prospective study (Local Ethical Committee No. 11792_BO_SK_2025) on April 4, 2025. The study was conducted in accordance with relevant guidelines and regulations.

## Funding

This research did not receive any specific grant from funding agencies in the public, commercial, or not-for-profit sectors.

## Availability of data and materials

All data generated or analysed during this study are included in this published article and its supplementary information files. The datasets used and analysed during the current study are available from the corresponding author on reasonable request.

## Declaration of competing interest

The authors declare that they have no competing interests.
