# Supplementary material for: Effects of increasing respiratory rate on ventilatory efficiency and mechanical costs during low-tidal-volume ventilation: a prospective physiological pilot study
Source: Ann Intensive Care. 2026 Jun 2;16:100098. doi: 10.1016/j.aicoj.2026.100098 (PMC13266209; doi:10.1016/j.aicoj.2026.100098)
Supplement: Supplementary file 1 [file mmc1.pdf]

Online Data Supplement

Effects of Increasing Respiratory Rate on Ventilatory Efficiency and Mechanical Costs during Low-Tidal-Volume Ventilation: A Prospective Physiological Pilot Study

Carolin Jung, Erietta Markou, Kai Florian Storch, Jona Wassong, Hans-Joerg Gillmann, Thomas Stueber

Table of Contents **page**

- **Figure S1. Schematic of the study procedure and stepwise respiratory rate protocol.** 3
- **Supplementary Methods** 4-14
- **Figure S2 Missing data pattern** 15
- **Figure S3. Patient Retention Across Respiratory Rate Steps** 16
- **Table S1. Reasons for Protocol Discontinuation by Protocol** 17
- **Table S2. Overview of measured and calculated parameters.** 18
- **Figure S4: Mechanical Power Components by Respiratory Rate.** 19
- **Figure S5. Simplified Power Index (4·ΔP+RR) by Rate** 20
- **Figure S6. Mechanical power trajectories across respiratory rate levels stratified by baseline ventilatory ratio** 21
- **Figure S7. Simplified Power Index (4·ΔP+RR) across respiratory rate levels stratified by baseline ventilatory ratio** 22
- **Figure S8. Observed $\dot{V}CO_2$ trajectories across respiratory rates, stratified by baseline ventilatory ratio.** 23
- **Table S3. Incremental CO_2 elimination efficiency across RR steps.** 24
- **Figure S9. Changes in arterial blood gas parameters** 25
- **Table S4A-B. Respiratory values across respiratory rate levels** 26-28
- **Table S5. Linear mixed-effects model results with FDR correction** 29-31

- **Table S6. Functional form comparison for all mixed-effects model outcomes** **32**
- **Figure S10. Residual diagnostics for the mechanical power and CO_2 elimination models with ventilatory ratio interaction.** **33**
- **Table S7. Linear mixed-effects model for mechanical power and carbon dioxide elimination ($\dot{V}CO_2$) with ventilatory ratio interaction.** **34**
- **Table S8. LMM results for marginal changes in ventilatory parameters.** **35**
- **Sensitivity analyses and Signal stability** **36**
- **Table S9. Summary of sensitivity analyses for MNAR assumptions** **37-39**
- **Figure S11A-G. Observed group means: Outcome trajectories across the full respiratory rate range.** **38-44**
- **Figure S12. Treatment Response Heterogeneity to Minute Ventilation Augmentation.** **45**
- **Table S10. Signal stability of volumetric capnography parameters across respiratory rate levels.** **46-47**
- **References** **47-48**

Figure S1. Schematic of the study procedure and stepwise respiratory rate protocol.

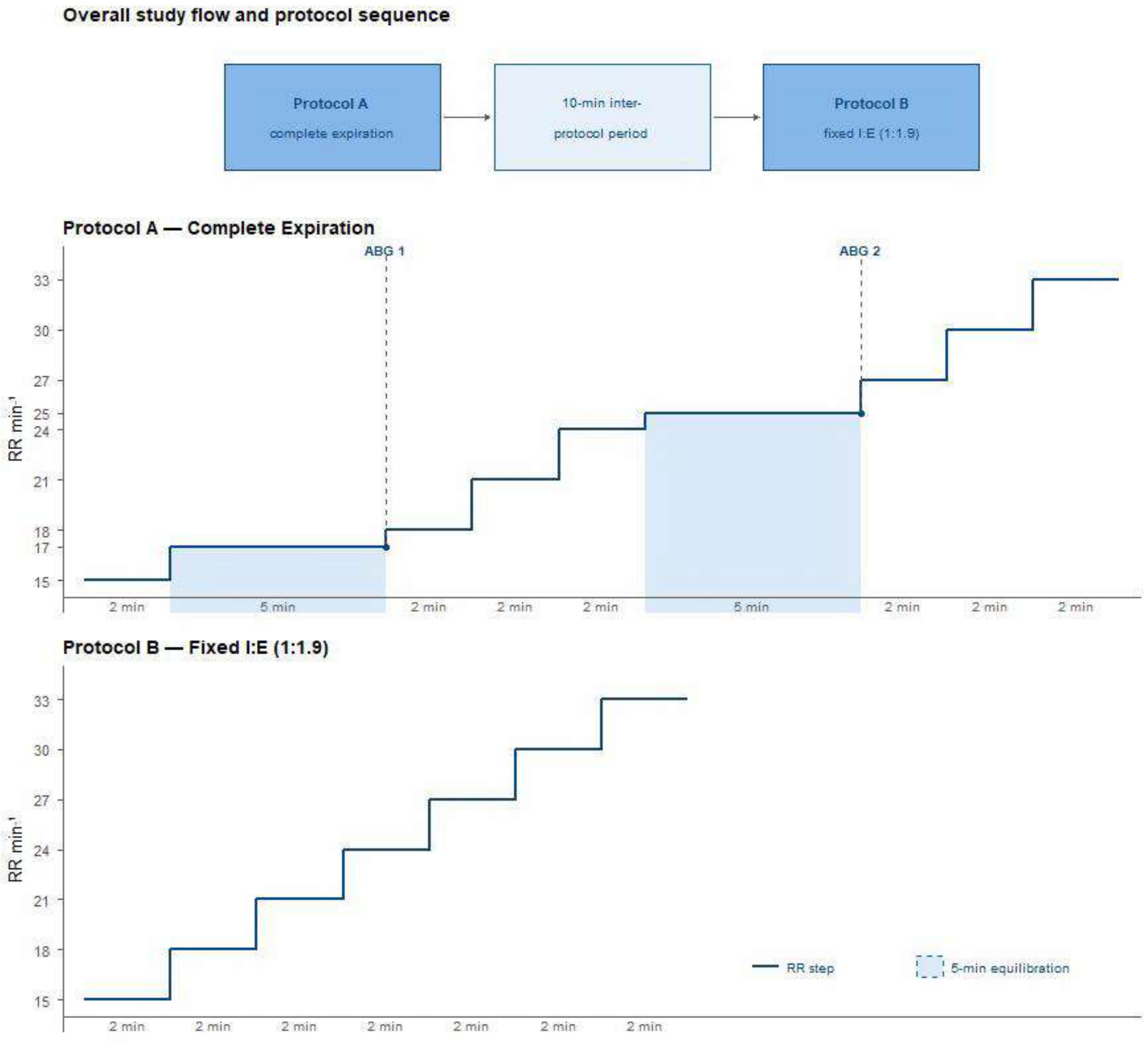

Each patient underwent two protocols in fixed order: Protocol A (complete expiration) followed by Protocol B (fixed I:E ratio 1:1.9), separated by a 10-minute inter-protocol equilibration period. Respiratory rate was increased stepwise by 3 min^{-1} from 15 to 33 min^{-1} and maintained for 2 minutes per step before five consecutive breath cycles were averaged. Within Protocol A, arterial blood gas samples were obtained at two predefined minute ventilation targets, 100 $mL \cdot kg^{-1}$ IBW (RR 17 min^{-1}) and 150 $mL \cdot kg^{-1}$ IBW (RR 25 min^{-1}), each preceded by a 5-minute equilibration period during which no ventilator settings were modified. Step height represents respiratory rate; shaded segments indicate equilibration periods prior to ABG sampling; ABG markers denote arterial blood gas sampling time points. RR, respiratory rate; ABG, arterial blood gas; IBW, ideal body weight; I:E, inspiratory-to-expiratory ratio.

Supplementary Methods

1. Ventilator Circuit Configuration

A single-limb (coaxial) breathing circuit was used for all measurements. To minimize and standardize apparatus dead space, all non-essential circuit components, including closed suction systems, in-line nebulizers, and flexible tubing extensions, were removed prior to the study protocol. A new heat and moisture exchanger (HME) filter was installed prior to each patient's measurement to ensure comparable dead space volume across all patients. The mainstream capnography sensor was positioned between the Y-piece and the HME, and the HME was connected directly to the endotracheal tube. The resulting total instrumental dead space inserted between the Y-piece and the endotracheal tube amounted to 41.3 mL, comprising the HME (Humid-Vent Filter Compact S, Teleflex Medical GmbH, Fellbach, Germany; 35 mL), the in-circuit flow sensor (Hamilton coaxial breathing circuit set with integrated flow sensor, 2.4 m, Hamilton Medical AG, Bonaduz, Switzerland; 1.3 mL), and the mainstream capnography sensor (CAPNOSTAT 5 Pediatric/Adult Airway Adapter, Philips Respironics, Murrysville, PA, USA; 5 mL). Endotracheal tube size was determined by the treating clinician according to clinical requirements.

2. Protocol A (Complete Expiration) – Detailed Algorithm

At each respiratory rate level, expiratory flow–time curves were continuously monitored on the ventilator display. Complete expiration was defined as expiratory flow returning to zero (±0.5 L/min) before the next inspiration, confirmed over at least three consecutive breaths. If this criterion was not met, the expiratory phase was incrementally extended in 0.1 steps of the I:E ratio until complete expiration was reached or a maximum ratio of 1:5 was attained. The corresponding I:E ratio was maintained for all subsequent measurements at that frequency. If complete expiration could not be achieved despite adjustment, the protocol was terminated at that frequency, and no further increments were performed.

3. Safety Stopping Criteria

The measurement protocol was terminated immediately if any of the following criteria were met: a reduction in end-tidal CO_2 exceeding 30% from baseline, a decrease in systolic blood pressure > 20%, a plateau pressure > 30 mbar, a driving pressure > 15 mbar, a tidal volume > 8 mL/kg IBW, or a peak inspiratory pressure ≥ 40 mbar. Reasons for protocol discontinuation are reported in Table S1. Stopping criteria were deliberately set conservatively to minimize the risk of harm due to the study intervention. The limits for tidal volume, plateau pressure, and driving pressure correspond to the upper bound of the lung-protective range recommended by current ARDS guidelines (1–3). The peak inspiratory pressure limit was chosen to avoid barotrauma and lies well below airway pressures associated with a substantially increased barotrauma risk (> 50 mbar (4)).

4. Steady-State Verification

To verify that the 2-minute equilibration period was sufficient to achieve steady-state conditions for capnographic measurements, within-subject coefficients of variation (CV) and normalised slopes were calculated for $\dot{V}CO_2$ as a primary gas exchange variable, and for VDaw and Phase III slope as variables derived from single-breath CO_2-volume curve analysis. P-values were adjusted for multiple comparisons using the Hochberg step-up procedure.

A different consideration applied to arterial blood gas sampling, where $PaCO_2$ equilibration after a step change in ventilation requires substantially longer than $\dot{V}CO_2$. The intervals between consecutive ABG measurements (median 20 min, IQR 18–24, with a dedicated 5-minute period of unchanged ventilator settings before each sample) were chosen to approximate near steady-state conditions while limiting patient exposure to high respiratory rates.

5. Capnographic Extrapolation to ABG Time Points

Because volumetric capnography measurements were obtained at predefined respiratory rate levels (15, 18, 21, 24, 27 min^{-1}), which did not coincide with the arterial blood gas sampling points (17 and 25 min^{-1}), patient-specific capnographic values at these intermediate frequencies were derived from quadratic mixed-effects models. For each capnographic variable required for the Enghoff calculation (Vte, $VeCO_2$, and VDaw), a separate model was fitted to data from the complete expiration protocol:

$$Y\_ij = \beta_0 + \beta_1 \cdot RR\_c + \beta_2 \cdot RR\_c^2 + u_{0i} + u_{1i} \cdot RR\_c + \varepsilon\_ij$$

where RR_c = RR − 15. Individual predictions at respiratory rates 17 and 25 min^{-1} were obtained incorporating both fixed and random effects to preserve patient-level variability. This approach leverages the full within-patient trajectory rather than relying on linear interpolation between adjacent measurement points and accounts for the non-linear relationships observed in several capnographic parameters.

6. Detailed Description of Calculated Variables

6.1 Respiratory monitoring and volumetric capnography

Ventilatory parameters were continuously recorded using the Hamilton C6 ventilator equipped with a mainstream volumetric capnography sensor. Directly measured parameters included tidal volumes (inspiratory and expiratory), airway pressures (peak inspiratory pressure, mean airway pressure, set PEEP), timing parameters (inspiratory and expiratory time), and flow rates (inspiratory and expiratory).

Volumetric capnography provided breath-by-breath measurements of carbon dioxide parameters, including end-tidal CO_2 partial pressure ($PetCO_2$), expiratory CO_2 volume per breath ($VeCO_2$), inspiratory CO_2 volume reflecting rebreathing ($ViCO_2$), total CO_2 elimination ($\dot{V}CO_2$), and the slope of Phase III of the volumetric capnogram ($SlopeCO_2$). Airway dead

space (VDaw) was determined by the ventilator using the Pre-Interface Expirate (PIE) method, which estimates the mean value of VDaw based on the differentiation of phase II in the volumetric capnogram (5).

6.2 Respiratory mechanics

Static respiratory mechanics were assessed at each respiratory rate level using standardized occlusion maneuvers. An inspiratory hold maneuver was performed to obtain plateau pressure (Pplat), and an expiratory hold maneuver was performed to measure total PEEP under static conditions (ExpHold). When expiratory hold measurements were unavailable, total PEEP was calculated as the sum of intrinsic PEEP and set PEEP:

$$PEEP\_total = ExpHold \text{ (if available), otherwise } PEEPi + PEEPe$$

Driving pressure (ΔP) was calculated as:

$$\Delta P = Pplat - PEEP\_total \text{ [mbar]}$$

Static respiratory system compliance (Cstat) was calculated as:

$$Cstat = Vte / \Delta P \text{ [mL/mbar]}$$

Inspiratory resistance (Rinsp) was calculated as:

$$Rinsp = (Ppeak - Pplat) / InspFlow \text{ [mbar·s/L]}$$

where inspiratory flow was converted from L/min to L/s. Of note, resistance values derived from decelerating flow patterns systematically underestimate true inspiratory resistance, as inspiratory flow at the time of peak pressure measurement is lower than mean inspiratory flow. Resistance values should therefore be interpreted with caution.

6.3 Dead space partitioning

Physiological dead space (VDphys) was calculated using the Enghoff modification of the Bohr equation:

$$VDphys = Vte \times (PaCO_2 - \bar{P}ECO_2) / PaCO_2 \ [mL]$$

The estimation of alveolar dead space (VDalv) was derived as:

$$VDalv = VDphys - VDaw \ [mL]$$

Dead space fraction was expressed as VD/VT = VDphys / Vte.

6.4 Gas exchange and ventilatory efficiency

The PaO_2/FiO_2 ratio was calculated as an index of oxygenation efficiency.

The expected $PaCO_2$ after respiratory rate augmentation was derived from the alveolar ventilation equation under the assumptions of constant dead space fraction and constant metabolic CO_2 production:

$$PaCO_2\_expected = PaCO_2\_baseline \times (MV_{100} / MV_{150})$$

where MV_{100} and MV_{150} denote the measured minute ventilations at target levels of 100 mL/kg IBW (respiratory rate 17 min^{-1}) and 150 mL/kg IBW (25 min^{-1}), respectively. Deviations between expected and observed $PaCO_2$ were quantified as the prediction error (observed $\Delta PaCO_2$ − expected $\Delta PaCO_2$).

Incremental CO_2 elimination efficiency was calculated between consecutive respiratory rate steps as $\Delta \dot{V}CO_2/\Delta MV$, expressed as millilitres of CO_2 eliminated per litre of additional minute ventilation, where MV = respiratory rate × Vte. The fractional efficiency ratio was calculated as $(\Delta \dot{V}CO_2/\dot{V}CO_2) / (\Delta MV/MV)$; a value of 1.0 indicates proportional response.

6.5 Mechanical power

Mechanical power was calculated using the simplified equation proposed by Gattinoni et al. and decomposed into its elastic, resistive, and static components:

$$MP = 0.1 \times RR \times VTi \times (Ppeak - 0.5 \times \Delta P)$$

$$MPstatic = 0.1 \times RR \times VTi \times PEEP\_total$$

$$MPelastic = 0.1 \times RR \times VTi \times 0.5 \times \Delta P$$

$$MPresistive = 0.1 \times RR \times VTi \times (Ppeak - Pplat)$$

The constant 0.1 converts from mbar × L to Joules. Multiplication by RR (breaths/min) yields J/min.

6.6 Simplified driving-pressure– and rate–based power index (4·ΔP+RR)

In addition to total mechanical power, we computed the simplified index 4·ΔP+RR proposed by Costa et al. (6) for each respiratory-rate step and protocol, to isolate the components most strongly associated with outcome. Driving pressure (ΔP) was calculated as the difference between plateau pressure and total PEEP under static conditions and expressed in mbar. Because the index is a dimensionless composite of pressure and rate, results are reported in index units.

7. Detailed Statistical Modeling

7.1 Mixed-effects model specification

To compare ventilation parameters between protocols across respiratory rates while accounting for the repeated-measures structure, mixed-effects models were fitted for all outcomes. The choice of mixed-effects modeling was motivated by: (1) within-subject correlation from multiple observations per patient; (2) expected inter-individual differences in

baseline values and responsiveness; and (3) natural handling of the unbalanced design from protocol discontinuation.

All models retained the same random-effect structure (patient-specific random intercept and slope for respiratory rate) and the same fixed-effects structure for protocol and protocol-by-respiratory-rate interactions, with quadratic terms included only in the quadratic specification. The functional form of the fixed-effect relationship between respiratory rate and outcome was selected for each outcome based on the Akaike Information Criterion (see Section 7.2).

The three candidate model specifications were:

Linear:

$$Y\_ij = \beta_0 + \beta_1 \cdot RR\_c + \beta_2 \cdot Protocol + \beta_3 \cdot (RR\_c \times Protocol) + u_{0i} + u_{1i} \cdot RR\_c + \varepsilon\_ij$$

Quadratic:

$$Y\_ij = \beta_0 + \beta_1 \cdot RR\_c + \beta_2 \cdot RR\_c^2 + \beta_3 \cdot Protocol + \beta_4 \cdot (RR\_c \times Protocol) + \beta_5 \cdot (RR\_c^2 \times Protocol) + u_{0i} + u_{1i} \cdot RR\_c + \varepsilon\_ij$$

Exponential (log-transformed outcome):

$$\log(Y\_ij) = \beta_0 + \beta_1 \cdot RR\_c + \beta_2 \cdot Protocol + \beta_3 \cdot (RR\_c \times Protocol) + u_{0i} + u_{1i} \cdot RR\_c + \varepsilon\_ij$$

where Y_ij denotes the outcome for patient i at observation j; RR_c is the centered respiratory rate (RR − 15); Protocol is a binary indicator (0 = Complete Expiration, 1 = Fixed I:E); β_0 is the intercept (expected value at RR 15 min^{-1} in the reference protocol); for the quadratic specification, β_1 and β_2 are the linear and quadratic effects of respiratory rate; β_3 is the main effect of protocol; β_4 and β_5 are the linear and quadratic interaction terms; for linear and exponential specifications, the corresponding β-coefficients represent the same effects without the quadratic terms; u_{0i} and u_{1i} are patient-specific random intercept and slope; and ε_ij is the residual error. Random effects were assumed to follow a multivariate normal distribution with unstructured covariance.

7.2 Selection of functional form

The functional form of the relationship between respiratory rate and outcome was selected for each outcome by comparing the three candidate specifications using the Akaike Information Criterion (AIC). The Bayesian Information Criterion (BIC) was reported as a sensitivity criterion. AIC and BIC for the exponential model (log-transformed outcome) were Jacobian-corrected to allow direct comparison on the original outcome scale. The functional form with the lowest AIC was retained as the primary specification for inference; the corresponding model coefficients are reported in Table S5, and the full functional-form comparison is provided in Table S6.

7.3 Interpretation of coefficients

For linear and quadratic models, β-coefficients represent the change in the outcome per unit predictor (e.g., per unit increase in respiratory rate). For exponential models, β-coefficients represent the change in log(Y) per unit predictor; the corresponding rate ratio (= exp(β)) provides a multiplicative interpretation on the original scale (e.g., a rate ratio of 1.10 corresponds to a 10 % increase per unit predictor). When marginal means or fitted trajectories were back-transformed to the original outcome scale for visualisation, Duan's smearing estimator was applied to correct for the bias introduced by simple exponentiation of log-scale predictions. Confidence intervals on the log scale were exponentiated to yield the corresponding interval on the original (multiplicative) scale. For quadratic specifications, a positive β_2 indicates an accelerating pattern (progressively steeper changes at higher respiratory rates), and a negative β_2 indicates a decelerating pattern or plateau formation. A significant RR_c^2 × Protocol interaction (β_5) indicates protocol-dependent curvature.

7.4 Estimated marginal means

Estimated marginal means (EMMs) were derived at each respiratory rate level (15, 18, 21, 24, 27 min^{-1}) from fitted models. EMMs represent population-level estimates averaged over the

random effects distribution. Pairwise comparisons between protocols were computed with 95% confidence intervals and Satterthwaite-adjusted p-values.

7.5 Incremental efficiency analysis

Incremental CO_2 elimination efficiency ($\Delta\dot{V}CO_2/\Delta MV$) was analysed using linear mixed-effects models with respiratory rate (centered at 15 min^{-1}), ventilation protocol, and their interaction as fixed effects and patient-specific random intercepts. Estimated marginal means with 95% confidence intervals were derived at each rate step.

7.6 Multiple testing correction

The primary endpoint (estimation of alveolar dead space) was tested at $\alpha = 0.05$ without correction. For exploratory outcomes analysed using mixed-effects models, outcomes were grouped into physiologically related families and the Benjamini–Hochberg procedure was applied within each family ($q < 0.05$):

- CO_2 elimination: $PetCO_2$, $\dot{V}CO_2$, $VeCO_2$, $ViCO_2$, Phase III Slope
- Dead space: VDaw
- Pressures: Ppeak, Pplat, Pmean, PEEPi, ΔP
- Volumes: VTi, VTe
- Timing and flow: TI, TE, InspFlow, ExpFlow
- Mechanical power: MP, MPelastic, MPresistive, MPstatic, 4·ΔP+RR

Both uncorrected p-values and FDR-adjusted q-values are reported for mixed-effects models.

7.7 VR-moderated models for mechanical power, 4·ΔP+RR and $\dot{V}CO_2$

To examine whether baseline ventilatory ratio (VR) moderated the rate-dependent response of mechanical power (MP), the simplified power index (*4·ΔP+RR)* and CO_2 elimination ($\dot{V}CO_2$), the corresponding mixed-effects models were extended to include baseline VR (centred at the cohort median) as a continuous moderator, with VR × RR and VR × RR^2 interaction terms. The functional form was selected by AIC and BIC as described in Section 7.2. For these two outcomes, model selection was complemented by inspection of residual plots and Q–Q diagnostics to confirm that the AIC-preferred specification satisfied distributional assumptions on the chosen scale (Figure S10).

Mechanical power was modelled on the log scale (AIC-preferred); coefficients therefore represent additive effects on log(MP) and multiplicative effects on the original scale via exp(β). $\dot{V}CO_2$ and *4·ΔP+RR* were modelled on the original scale with linear and quadratic RR terms and their VR interactions retained per AIC. Coefficients with 95% confidence intervals are reported in Table S7.

8. Missing Data and Sensitivity Analyses

Discontinuation of the measurement protocol due to predefined safety criteria resulted in missing data at higher respiratory rates. The primary analysis was restricted to respiratory rates ≤27 min^{-1}, where patient retention remained ≥80% in both protocols. For transparency, observed group means with 95% confidence intervals across all respiratory rate levels (15–33 min^{-1}) using all available observations at each level (available-case analysis) are reported in Figures S11A–G, with acknowledgment that results at RR >27 min^{-1} represent a selected subpopulation.

Sensitivity analyses were performed separately for key safety parameters (Ppeak, PEEPi, $PetCO_2$) to assess the impact of informative censoring at respiratory rates above the primary analysis cutoff:

(a) Bounds analysis: Missing values were imputed under increasingly pessimistic scenarios, ranging from baseline-stable (last observed value carried forward) to 100% above baseline.

(b) Pattern mixture models: Models were fitted with delta-adjustment (δ = 0 to 50 units) applied to LOCF-imputed values for non-completers.

(c) Tipping point analysis: The magnitude of δ required to either change statistical significance (p crossing 0.05) or push estimates beyond pre-specified thresholds of clinical relevance (>20% change from baseline) was determined.

9. Treatment Response Heterogeneity

To explore heterogeneity in treatment response, patients were stratified by lung health status into those without an active pulmonary condition (postoperative patients with healthy lungs) and those with an acute pulmonary condition (ARDS, pneumonia, obesity-related respiratory impairment, or obstructive pulmonary disease). Between-group comparisons were conducted using independent t-tests or Mann–Whitney U tests according to Shapiro–Wilk normality testing. Within-group changes were analyzed with paired t-tests or Wilcoxon signed-rank tests. Correlations between baseline ventilatory ratio and treatment outcomes were assessed using Pearson's r or Spearman's ρ as appropriate.

Patients were additionally stratified by median baseline ventilatory ratio into high and low VR groups to assess whether baseline gas exchange impairment modulated treatment response.

These subgroup analyses were exploratory, unadjusted for multiple comparisons, and should be interpreted as hypothesis-generating.

Figure S2. Missing data pattern across respiratory parameters and respiratory rates.

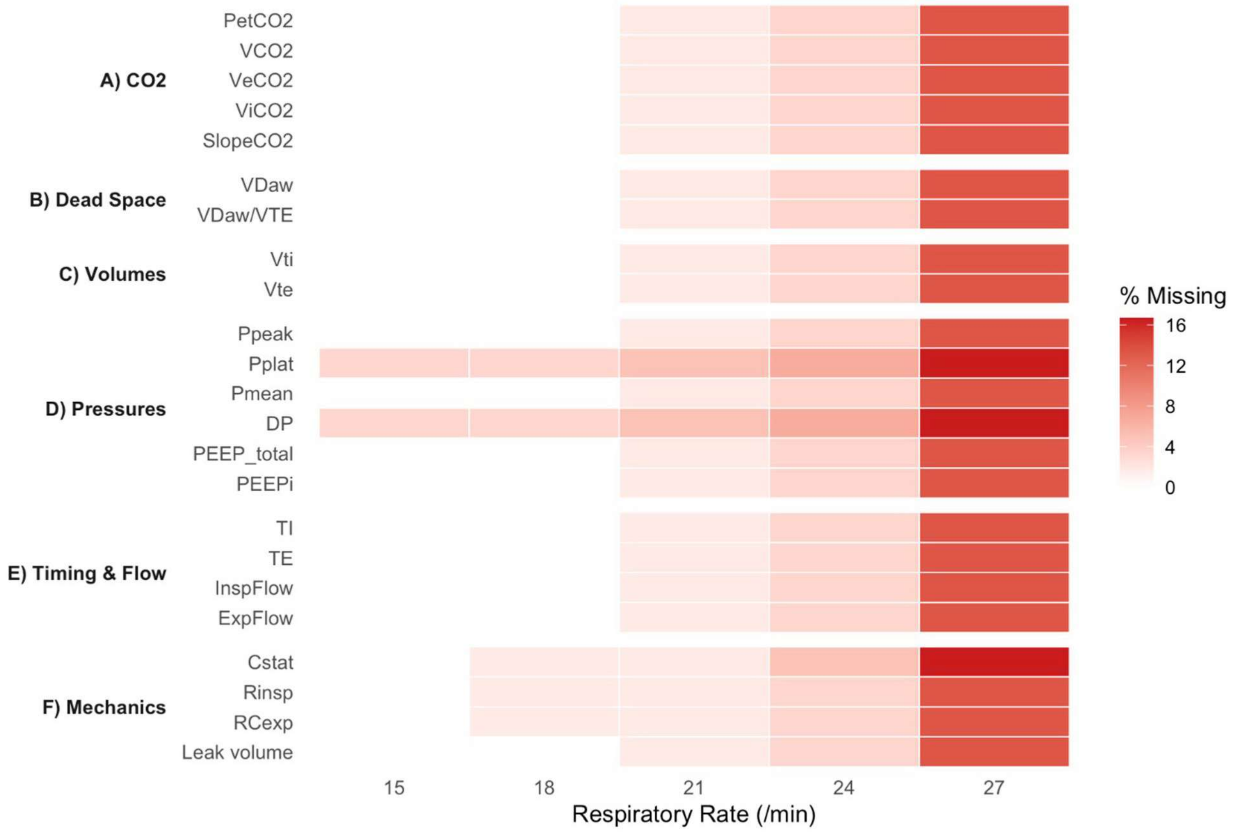

Heatmap displaying the percentage of missing observations for each of 29 respiratory parameters (rows) at each respiratory rate level (columns). Color intensity indicates the proportion of missing data, ranging from white (0% missing, complete data) to dark red (≥ 60% missing).

Figure S3. Patient Retention Across Respiratory Rate Steps.

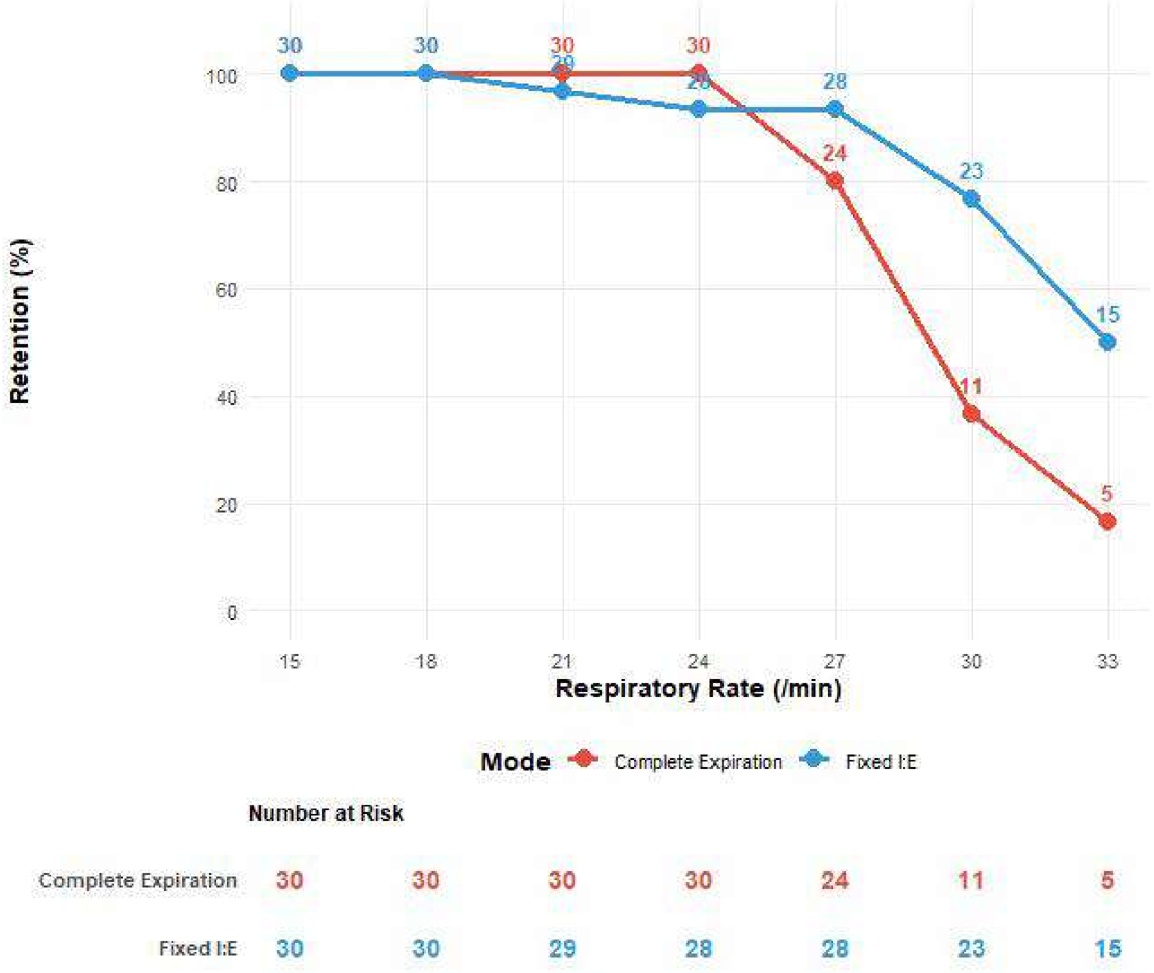

Retention rates comparing both Complete Expiration (red) and Fixed I:E ratio (blue) protocols. Numbers indicate patients with valid measurements at each step. *Abbreviations: I:E, inspiratory-to-expiratory ratio.*

Table S1. Reasons for Protocol Discontinuation by Protocol

Discontinuation reason	Complete exhalation	Fixed I:E
Driving Pressure > 15cm	1	0
Incomplete Expiration	13	0
Ppeak ≥40	5	0
$PetCO_2$ decrease > 30%	6	13

Safety-related discontinuations stratified by ventilation protocol. Complete Expiration protocol discontinuations were predominantly due to respiratory mechanics limitations (incomplete expiration, n=13; elevated airway pressures, n=6), whereas Fixed I:E protocol discontinuations were exclusively due to too strong decline of $PetCO_2$. Some longitudinal series showed mid-protocol missing values due to technical difficulties, necessitating exclusion of all subsequent measurements per patient/protocol for longitudinal analysis. Two such discontinuations occurred in Fixed I:E protocol. *Abbreviations: $PetCO_2$, partial pressure of end-tidal carbon dioxide; Ppeak, peak inspiratory pressure; I:E, inspiratory-to-expiratory ratio.*

Table S2. Overview of measured and calculated parameters.

Category	Parameter	Unit	Source	Description
**CO_2 Parameters**	$PetCO_2$	mmHg	Ventilator-derived	End-tidal CO_2 partial pressure
	$\dot{V}CO_2$	mL/min	Ventilator-derived	Total CO_2 elimination per minute
	$VeCO_2$	mL	Ventilator-derived	Expiratory CO_2 volume per breath
	$ViCO_2$	mL	Ventilator-derived	Inspiratory CO_2 volume per breath
	Phase III slope	mmHg/L	Ventilator-derived	Phase III slope (ventilation homogeneity)
**Dead Space**	VDaw	mL	Ventilator-derived	Airway dead space (Fowler method)
	VDphys	mL	Calculated	Physiological dead space (Enghoff equation)
	VDalv	mL	Calculated	Alveolar dead space (VD_Enghoff − VDaw)
**Volumes**	VTi	mL	Ventilator-derived	Inspiratory tidal volume
	VTe	mL	Ventilator-derived	Expiratory tidal volume
**Pressures**	Ppeak	mbar	Ventilator-derived	Peak inspiratory pressure
	Pplat	mbar	Manually measured	Plateau pressure
	Pmean	mbar	Ventilator-derived	Mean airway pressure
	PEEPi	mbar	Ventilator-derived	Intrinsic PEEP
	$PEEP_{total}$	mbar	Manually measured or calculated	Total PEEP (expiratory hold or PEEPi + PEEPe)
	ΔP	mbar	Calculated	Driving pressure (Pplat – $PEEP_{total}$)
**Timing**	TI	s	Ventilator-derived	Inspiratory time
	TE	s	Ventilator-derived	Expiratory time
**Flows**	InspFlow	L/min	Ventilator-derived	Mean inspiratory flow
	ExpFlow	L/min	Ventilator-derived	Mean expiratory flow
**Mechanics**	Cstat	mL/mbar	Calculated	Static compliance (Vte / ΔP)
	Rinsp	mbar·s/L	Calculated	Inspiratory resistance ([Ppeak − Pplat] / Flow)
	RCexp	s	Ventilator-derived	Expiratory time constant
	Leak volume	mL	Ventilator-derived	Leak volume
**Gas Exchange**	P/F ratio	mmHg	Calculated	PaO_2/FiO_2 ratio
	VR	—	Calculated	Ventilatory ratio
**Mechanical Power**	MP, MPstatic, MPelastic, MPresistive	J	Calculated	Mechanical Power
	4·ΔP+RR	—	Calculated	Simplified power index

Parameters are grouped by category. Source indicates whether the parameter was directly measured by the ventilator or calculated from measured values. Calculated parameters are derived using the formulas described in the section 2.

Figure S4: Mechanical Power Components by Respiratory Rate.

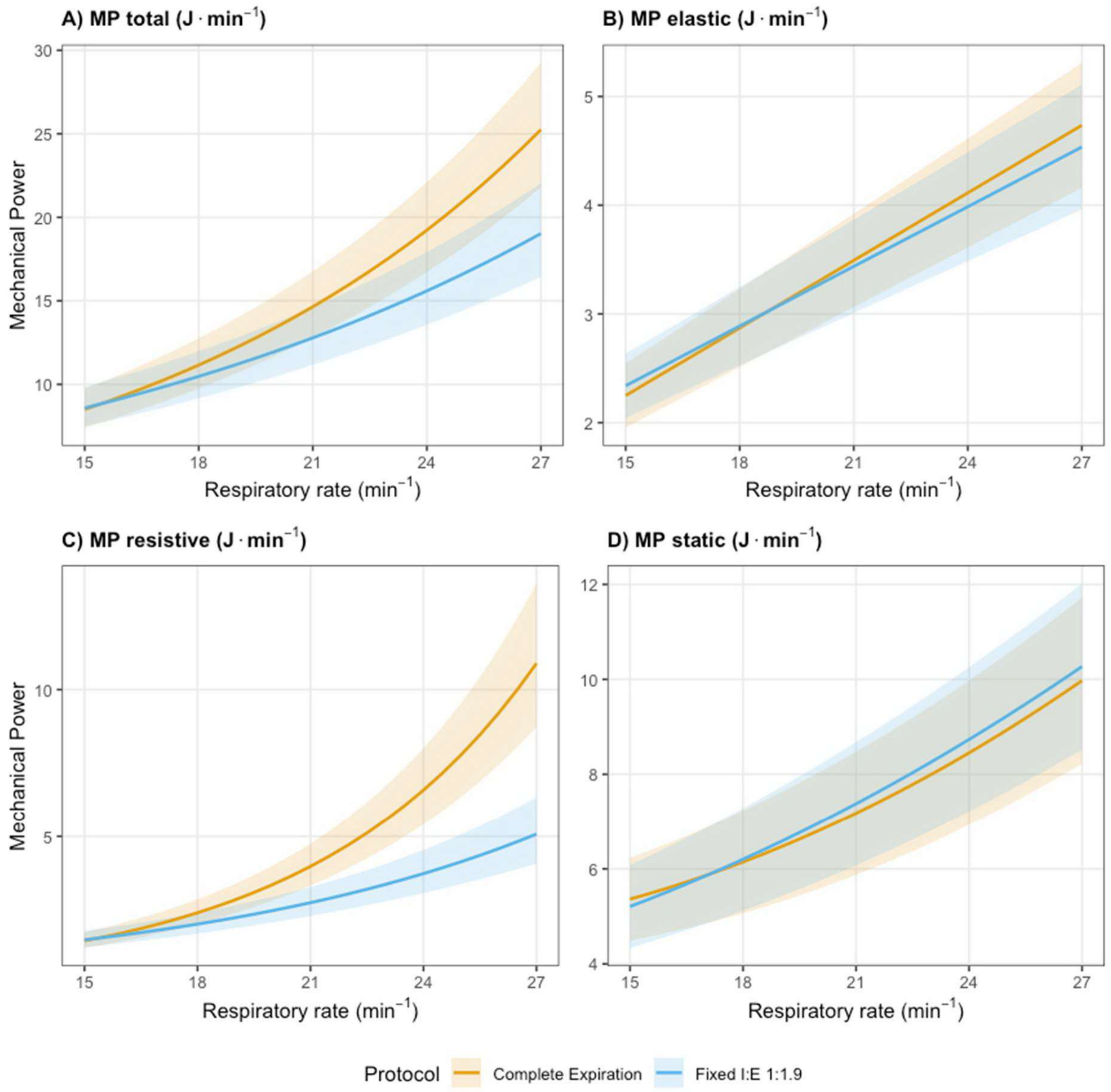

Estimated marginal means (95% CI) derived from linear mixed-effects models with outcome-specific functional forms (A: log-linear; B: linear; C: log-linear; D: quadratic). Complete Expiration protocol (orange) vs Fixed I:E protocol (blue). A) Total mechanical power exhibits pronounced non-linear increase under complete expiration with significant protocol divergence above RR 21. B) Elastic component increases linearly and equivalently under both protocols. C) Resistive component drives the protocol divergence, with marked acceleration under complete expiration. D) The static component shows parallel non-linear trajectories with marginally higher values under fixed I:E. Analysis based on n=30 subjects with 279–289 repeated observations across RR 15–27 min^{-1}.

Figure S5. Simplified Power Index (4·ΔP+RR) by Rate

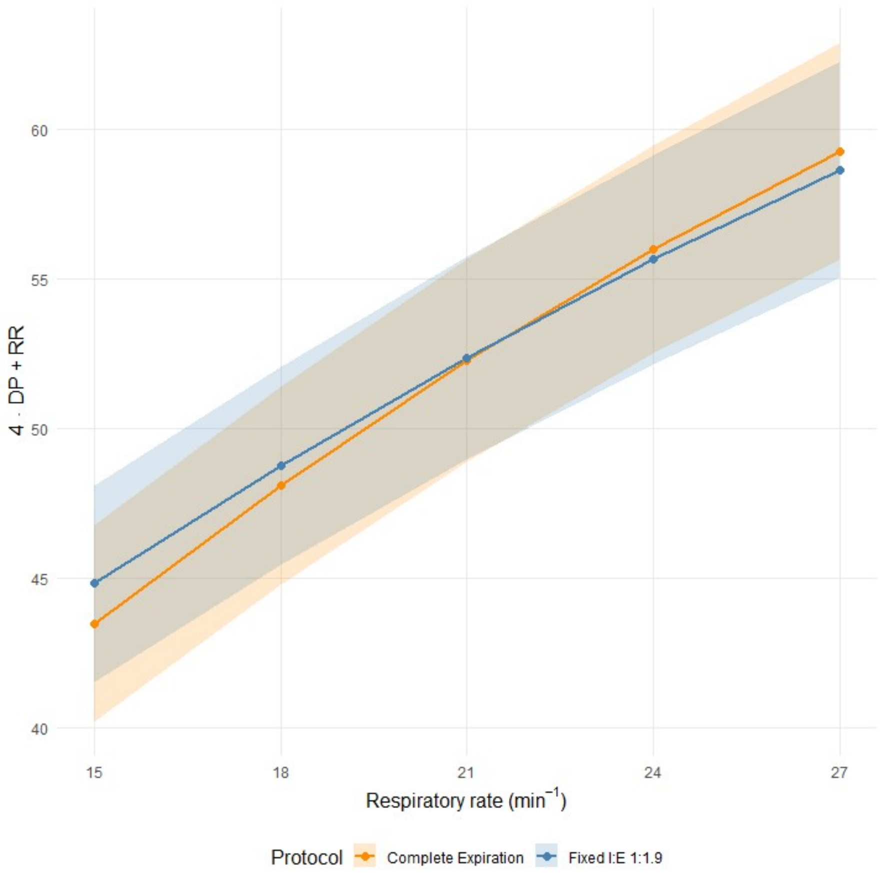

Estimated marginal means (95% CI) derived from linear mixed-effects models (quadratic). Complete Expiration protocol (orange) vs Fixed I:E protocol (blue). To isolate the power components most strongly associated with outcome, we additionally modelled the simplified, mortality-validated index 4·ΔP+RR. The index increased approximately linearly with rate (β = 1.62 index units·min^{-1}, $p < 0.001$), with only slight deceleration at higher rates (quadratic β = −0.025, $p = 0.049$). Estimated marginal means rose from 43.5 to 59.3 under complete expiration (+36%) and from 44.8 to 58.7 under fixed I:E (+31%) across RR 15 to 27 min^{-1}, which is far less than the corresponding increases in total mechanical power (+181% and +122%). In contrast to total mechanical power, which showed pronounced protocol-specific divergence, the rate response of the 4·ΔP+RR index did not differ between protocols (RR × protocol $p = 0.23$; RR^2 × protocol $p = 0.65$).

Figure S6. Mechanical power trajectories across respiratory rate levels stratified by baseline ventilatory ratio

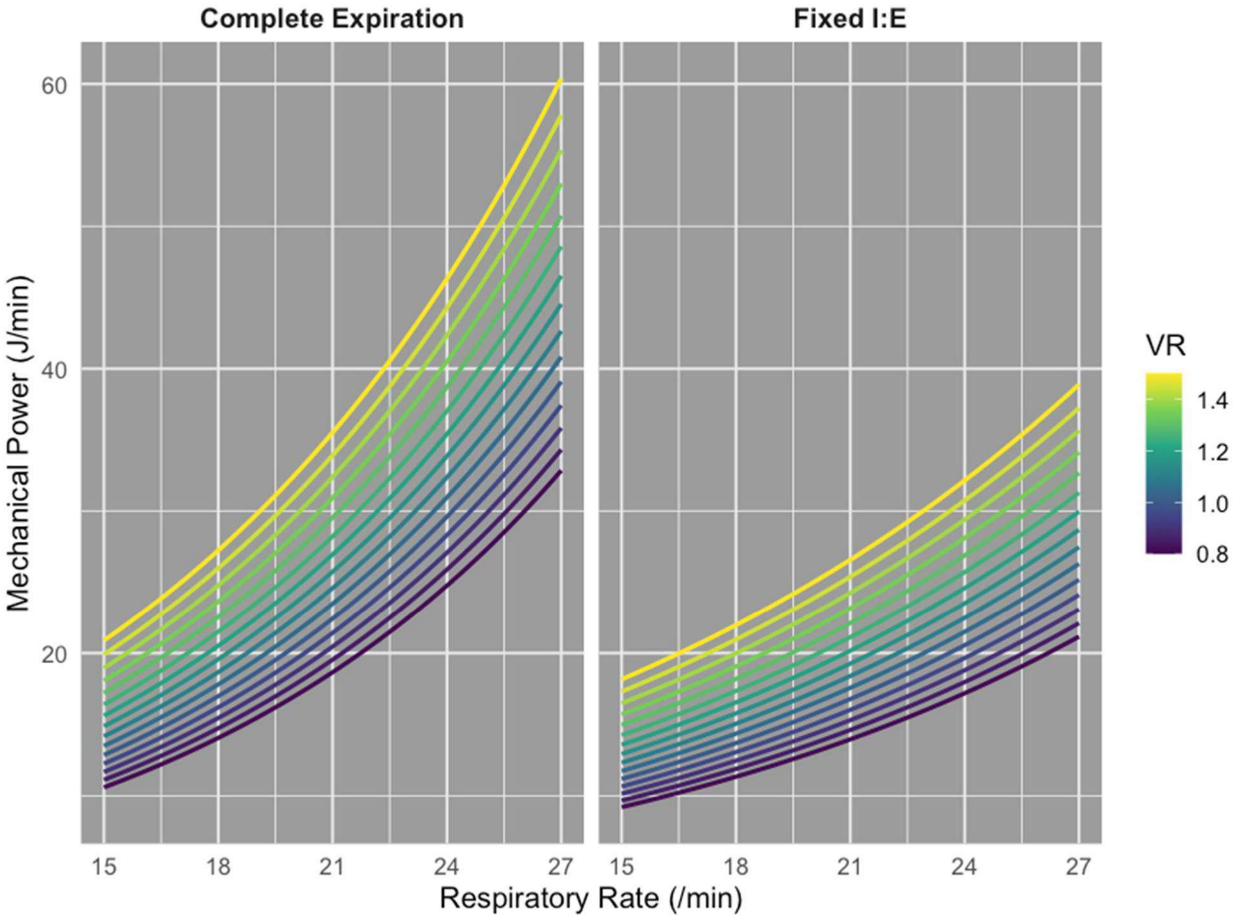

Predicted mechanical power (MP, J·min⁻¹) under Protocol A: complete expiration (left) and Protocol B: fixed I:E (1:1.9, right). Each line represents the predicted trajectory for a given baseline ventilatory ratio (VR), ranging from 0.80 (dark) to 1.50 (light) in 0.05 increments. Predictions derive from a linear mixed-effects model fitted to log-transformed MP, with respiratory rate (mean-centred), protocol, baseline VR (mean-centred), and the RR × Protocol and RR × VR interactions as fixed effects, and a random intercept and RR slope per subject. Fixed-effect predictions were back-transformed to the original scale using Duan's smearing estimator to correct for retransformation bias. Analysis restricted to respiratory rates 15–27 min⁻¹.

Figure S7. Simplified Power Index (4·ΔP+RR) across respiratory rate levels stratified by baseline ventilatory ratio

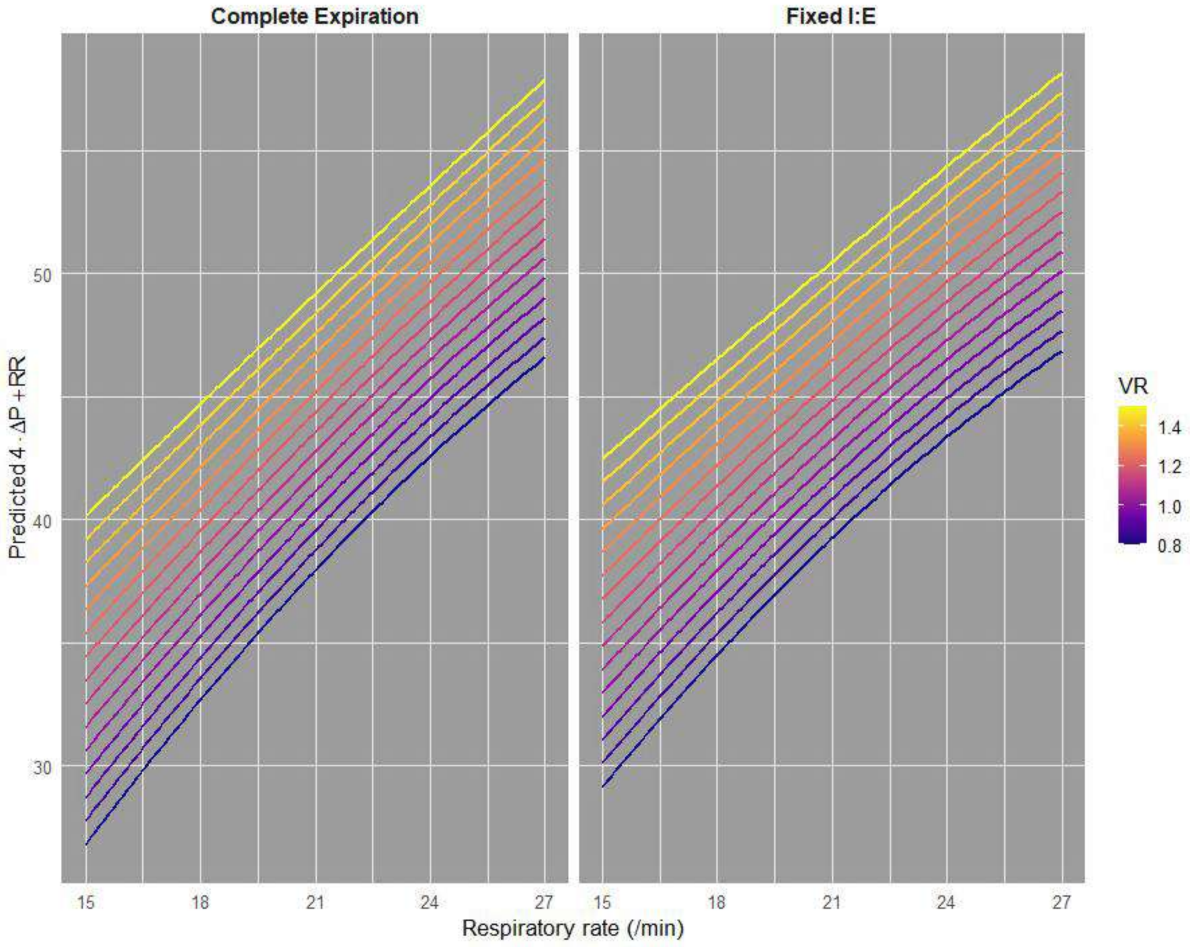

4·ΔP+RR index under Protocols A (left) Protocol B (right). Each line represents the predicted trajectory for a given baseline ventilatory ratio (VR), ranging from 0.80 (blue) to 1.50 (yellow) in 0.05 increments. Predictions derive from a linear mixed-effects model fitted to 4·ΔP + RR, with respiratory rate (mean-centred), protocol, baseline VR (mean-centred), and the RR × Protocol and RR × VR interactions as fixed effects, and a random intercept and RR slope per subject. Analysis restricted to respiratory rates 15–27 min^{-1}. Baseline ventilatory ratio did not moderate the rate trajectory of the 4·ΔP + RR index (RR × VR $p = 0.63$; RR^2 × VR $p = 0.28$). Baseline VR was associated only with the overall level of the index (β = 16.0 index units per VR unit, $p = 0.026$), consistent with the higher baseline driving pressure in patients with elevated VR.

Figure S8. Observed $\dot{V}CO_2$ trajectories across respiratory rates, stratified by baseline ventilatory ratio.

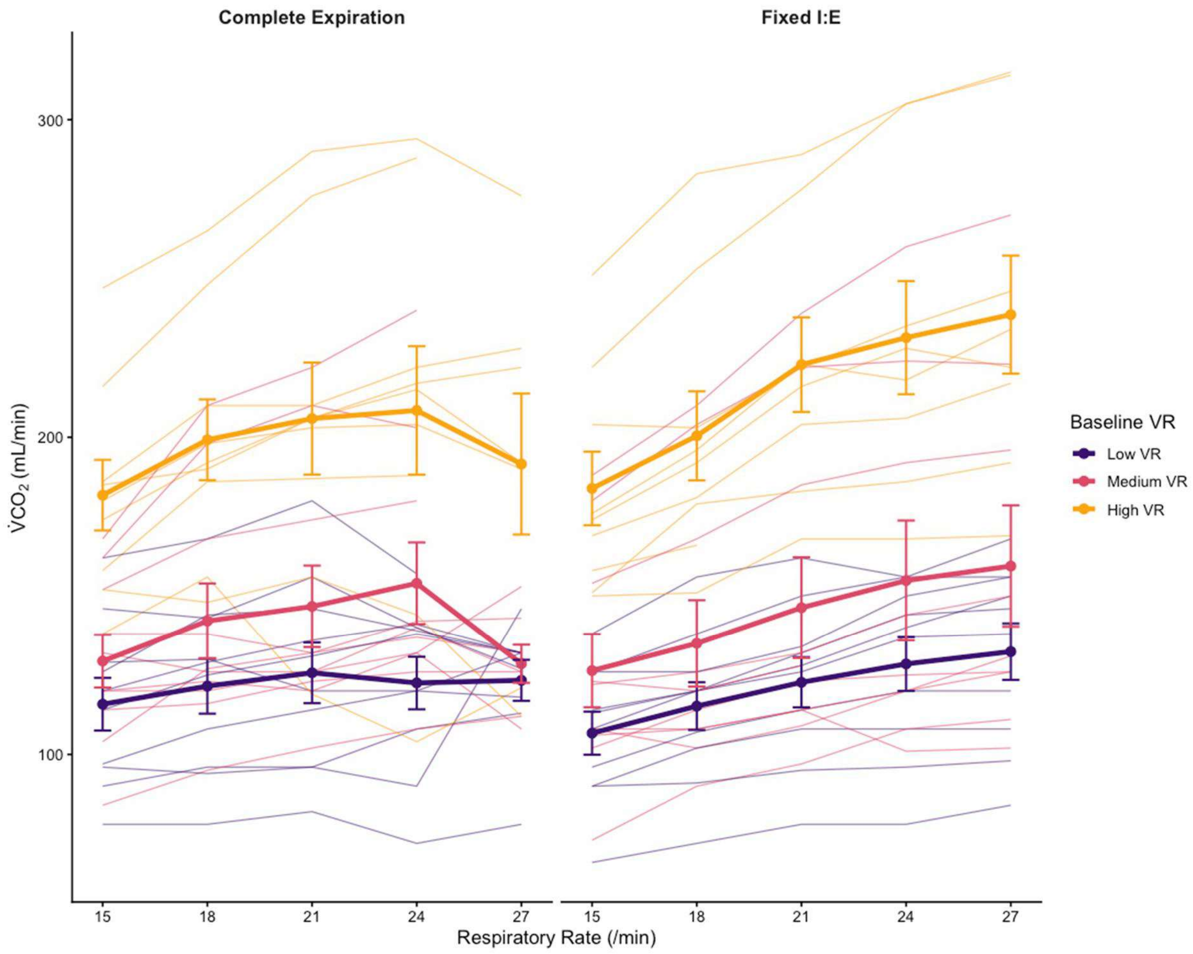

Individual patient trajectories (thin lines) and group means ± standard error of the mean (bold lines, error bars) of CO_2 elimination ($\dot{V}CO_2$) across respiratory rate steps (15–27 min^{-1}), stratified by tertiles of baseline ventilatory ratio (Low VR, Medium VR, High VR), under the Protocol A (left) and B (right). Under complete expiration, mean $\dot{V}CO_2$ in the High-VR tertile peaked between 21 and 24 min^{-1} and declined at 27 min^{-1}, consistent with an inverted-U pattern, whereas Low- and Medium-VR tertiles showed continued, more gradual increases. With fixed I:E, $\dot{V}CO_2$ continued to increase across rate steps in all VR tertiles, with a slight attenuation of the slope between 24 and 27 min^{-1} in the High-VR tertile. n = 29 patients with available baseline ventilatory ratio data.

Table S3. Incremental CO_2 elimination efficiency across respiratory rate steps.

RR Step (min^{-1})	Overall (95% CI)	Complete Expiration (95% CI)	Fixed I:E 1:1.9 (95% CI)
15→18	8.1 (6.2 to 10.1)	7.6 (4.9 to 10.2)	8.7 (6.0 to 11.3)
18→21	5.9 (4.4 to 7.4)	4.7 (2.9 to 6.6)	7.1 (5.2 to 9.0)
21→24	3.7 (2.2 to 5.2)	1.9 (−0.1 to 3.8)	5.5 (3.6 to 7.4)
24→27	1.5 (−0.5 to 3.6)	−1.0 (−3.8 to 1.9)	4.0 (1.3 to 6.7)

Estimated marginal means (95% CI) from a linear mixed-effects model with respiratory rate and ventilation protocol as fixed effects and patient-specific random intercepts. Values represent millilitres of additional CO_2 eliminated per litre of additional minute ventilation ($\Delta\dot{V}CO_2/\Delta MV$) at each consecutive respiratory rate step. Overall estimates are averaged across both protocols. n = 30 patients, 229 observations.

Figure S9. Changes in arterial blood gas parameters following increased minute ventilation.

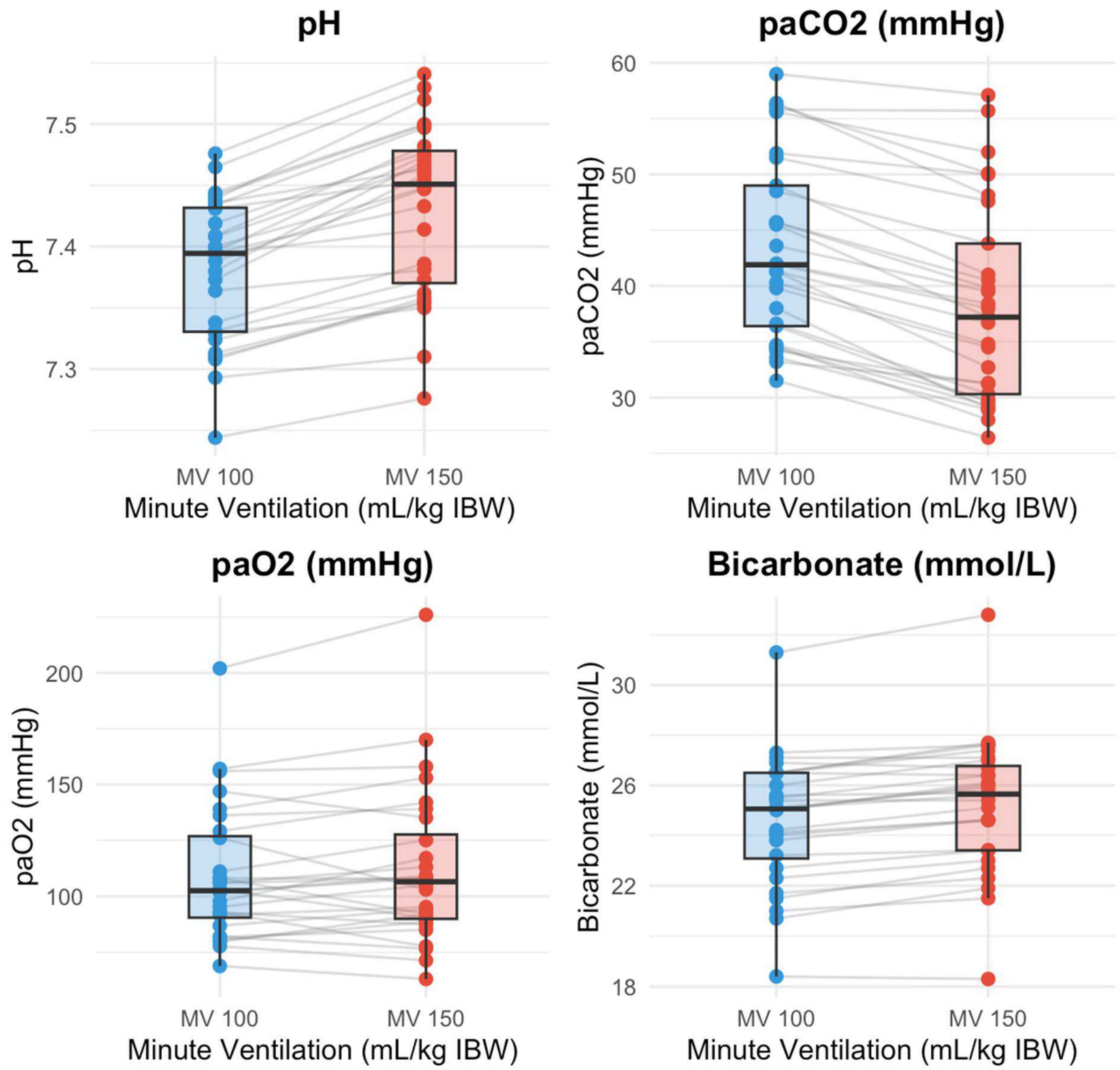

Paired plots showing individual patient trajectories (gray lines) and group distributions (boxplots) for pH (A), $PaCO_2$ (B), PaO_2 (C), and bicarbonate (D) at baseline minute ventilation (MV 100 mL/kg IBW, blue) and after increasing minute ventilation by 50% through respiratory rate augmentation (MV 150 mL/kg IBW, red). Tidal volume remained fixed at 6 mL/kg IBW. Each point represents an individual measurement; connecting lines indicate paired observations from the same patient (n = 29). Boxplots display median (horizontal line), interquartile range (box), and range (whiskers). Increasing minute ventilation resulted in a significant rise in pH (7.38 ± 0.06 to 7.43 ± 0.07, $p < 0.001$) and decrease in $PaCO_2$ (43.4 ± 8.3 to 38.2 ± 8.8 mmHg, $p < 0.001$), while PaO_2 and bicarbonate remained largely unchanged.

Table S4A. Respiratory values across respiratory rate levels in Complete Expiration Protocol

Category	Outcome	Unit	RR 15	RR 18	RR 21	RR 24	RR 27	Δ%
A) CO2 Parameters	$PetCO_2$	mmHg	42 ± 8.2	41.1 ± 9.0	38.7 ± 8.7	36.2 ± 8.7	32.7 ± 7.8	-22.2%
	$\dot{V}CO_2$	ml/min	141.7 ± 39.2	153.33 ± 46.1	157.9 ± 51.9	160.1 ± 54.5	146.3 ± 45.1	+3.3%
	$VeCO_2$	ml	11.2 ± 3.1	10.9 ± 3.3	10.3 ± 3.2	9.5 ± 2.9	8.3 ± 2.2	-26.2%
	$ViCO_2$	ml	1.8 ± 1.1	2.2 ± 1.0	2.6 ± 1.1	2.8 ± 1.2	2.8 ± 1.1	+60.4%
	Phase III Slope	mmHg/L	5.6 ± 3	5.55 ± 3.1	5.6 ± 3	5.6 ± 3	5.6 ± 2.9	-0.1%
B) Dead Space	VDaw	ml	122.9 ± 25.1	122.8 ± 28.3	119 ± 27.7	111.4 ± 27.3	103.6 ± 29.4	-15.7%
C) Volumes	VTi	ml	427.6 ± 69.4	426.4 ± 72.2	429.8 ± 74.5	433.4 ± 75	423.2 ± 71.7	-1%
	VTe	ml	405.7 ± 60.2	403.7 ± 66.4	406.9 ± 67.4	408.8 ± 66.4	394.6 ± 59.1	-2.7%
D) Pressures	Ppeak	mbar	18.2 ± 5	19.2 ± 5.5	21.1 ± 6.1	23.9 ± 6.5	27.1 ± 7.3	+49.1%
	Pplat	mbar	15.3 ± 4.7	15.4 ± 4.9	15.6 ± 4.7	15.8 ± 4.8	15.8 ± 4.8	+3.1%
	Pmean	mbar	10.6 ± 3.6	10.8 ± 3.8	11.2 ± 3.9	11.4 ± 3.7	11.5 ± 3.6	+8%
	ΔP	mbar	7.1 ± 2.1	7.6 ± 2.4	7.8 ± 2.3	7.9 ± 2.4	8.1 ± 2.5	+14.2%
	PEEPtotal	mbar	8.3 ± 3.3	7.8 ± 3.3	7.9 ± 3.3	8 ± 3.3	7.9 ± 3.2	-5.6%
	PEEPi	mbar	0.5 ± 0.4	0.5 ± 0.3	0.5 ± 0.3	0.5 ± 0.4	0.6 ± 0.6	+2.1%
E) Timing & Flow	TI	s	1.3 ± 0.1	1.1 ± 0.1	0.8 ± 0.1	0.6 ± 0.1	0.5 ± 0.1	-62.9%
	TE	s	2.7 ± 0.1	2.3 ± 0.1	2 ± 0.1	1.9 ± 0.1	1.7 ± 0.1	-34.7%
	InspFlow	L/min	28.6 ± 8.4	35.7 ±15.5	43.6 ± 15.8	57.5 ± 24	69.4 ± 26.3	+142.8%
	ExspFlow	L/min	34.4 ± 6.8	35.1 ± 7.0	36.1 ± 6.7	38.2 ± 10.9	38.2 ± 5.9	+11.3%
F) Mechanics	Cstat	ml/mbar	62.9 ± 25.3	56.8 ± 18.7	55.9 ± 18.2	55.3 ± 18.4	53.3 ± 20.6	-15.3%
	Rinsp	mbar·s/L	6.1 ± 3.5	6.5 ± 3.6	7.2 ± 3.5	8.1 ± 3.5	9.5 ± 4.6	+55.7%
	RCexsp	s	0.7 ± 0.2	0.65 ± 0.16	0.6 ± 0.1	0.6 ± 0.1	0.6 ± 0.1	-15.1%
	Leak volume	ml	4.8 ± 3.8	5.2 ± 3.5	5.2 ± 4	5.5 ± 4.2	6.5 ± 4.5	+34.5%
G) Mechanical Power	MP	J/min	9.3 ± 3.3	11.7 ± 4.6	15.5 ± 6.8	20.8 ± 9.2	26.1 ± 10.7	+180.4%
	MPelastic	J/min	2.2 ± 0.8	2.9 ± 1.1	3.5 ± 1.2	4.1 ± 1.4	4.5 ± 1.5	+102.1%
	MPstatic	J/min	5.4 ± 2.4	6.1 ± 2.9	7.2 ± 3.5	8.4 ± 4.1	9.1 ± 4.2	+69.3%
	MPresist	J/min	1.8 ± 1.1	2.8 ± 2.3	4.9 ± 4	8.5 ± 6	12.7 ± 8.1	+623.3%

Table 4B. Respiratory values across respiratory rate levels in Fixed I:E Protocol

Category	Outcome	Unit	RR 15	RR 18	RR 21	RR 24	RR 27	Δ%
A) CO2 Parameters	$PetCO_2$	mmHg	41.2 ± 10	40.0 ± 10.0	37.9 ± 9.7	35.8 ± 9.6	33.4 ± 8.9	-18.8%
	$\dot{V}CO_2$	ml/min	137.9 ± 44	148.6 ± 50.2	159.6 ± 56.1	167.3 ± 60.7	172.3 ± 62.6	+25%
	$VeCO_2$	ml	11 ± 3.6	10.5 ± 3.5	10 ± 3.4	9.5 ± 3.2	8.8 ± 3	-19.8%
	$ViCO_2$	ml	1.7 ± 1.1	2.1 ± 0.9	2.2 ± 0.9	2.3 ± 0.9	2.3 ± 0.9	+39.3%
	Phase III Slope	mmHg/L	5.5 ± 3.3	5.5 ± 3.1	5.3 ± 2.9	5.2 ± 2.8	4.9 ± 2	-11.2%
B) Dead Space	VDaw	ml	122.5 ± 26.7	121.6 ± 27.1	119.5 ± 26.7	120.9 ± 29	121.2 ± 37.3	-1.1%
C) Volumes	VTi	ml	421.4 ± 70.1	422.4 ± 70.7	424.7 ± 71.4	431.2 ± 71.3	430.7 ± 72.9	+2.2%
	VTe	ml	396.5 ± 60.1	400.1 ± 64.8	402.3 ± 66.4	408.6 ± 65.7	408 ± 66.7	+2.9%
D) Pressures	Ppeak	mbar	18.2 ± 5.3	18.8 ± 5.3	19.4 ± 5.6	20.6 ± 5.9	21.5 ± 5.8	+18.1%
	Pplat	mbar	15.6 ± 4.7	15.6 ± 4.7	15.8 ± 4.7	16.3 ± 4.8	16.6 ± 4.7	+6.2%
	Pmean	mbar	10.6 ± 3.7	10.8 ± 3.8	11.1 ± 3.8	11.7 ± 4	12.2 ± 4.1	+15.8%
	ΔP	mbar	7.4 ± 2.2	7.8 ± 2.2	7.8 ± 2.3	7.8 ± 2.4	7.9 ± 2.3	+6.9%
	PEEPtotal	mbar	8.2 ± 3.3	7.9 ± 3.3	8.1 ± 3.4	8.5 ± 3.4	8.7 ± 3.3	+6.3%
	PEEPi	mbar	0.5 ± 0.2	0.6 ± 0.2	0.7 ± 0.3	1 ± 0.5	1.3 ± 0.6	+137.9%
E) Timing & Flow	TI	s	1.4 ± 0	1.2 ± 0.0	1 ± 0	0.9 ± 0	0.8 ± 0	-44.2%
	TE	s	2.6 ± 0	2.2 ± 0.0	1.9 ± 0	1.6 ± 0	1.5 ± 0	-44%
	InspFlow	L/min	27.6 ± 8.1	31.4 ± 7.5	35.2 ± 7.5	39.5 ± 7.7	43.2 ± 8.2	+56.7%
	ExspFlow	L/min	35.7 ± 6.5	35.8 ± 6.1	36.5 ± 6.3	40.2 ± 17.8	38.1 ± 6	+6.7%
F) Mechanics	Cstat	ml/mbar	57.4 ± 18	54.5 ± 16.5	55.6 ± 18.7	56.8 ± 21.3	55.4 ± 18.9	-3.4%
	Rinsp	mbar·s/L	5.8 ± 2.6	6.1 ± 2.7	6.2 ± 2.5	6.5 ± 2.9	6.9 ± 2.6	+18.6%
	RCexsp	s	0.6 ± 0.2	0.6 ± 0.1	0.6 ± 0.1	0.6 ± 0.1	0.6 ± 0.1	-9.3%
	Leak volume	ml	5.1 ± 3.6	5.2 ± 3.4	5 ± 3.2	5.1 ± 3.6	5 ± 3.9	-1.9%
G) Mechanical Power	MP	J/min	9.1 ± 3.5	11.2 ± 4.2	13.7 ± 5.3	17.1 ± 6.6	20.2 ± 7.7	+122.7%
	MPelastic	J/min	2.3 ± 0.8	2.9 ± 0.9	3.4 ± 1.1	4 ± 1.3	4.5 ± 1.5	+96.3%
	MPstatic	J/min	5.3 ± 2.4	6.1 ± 2.9	7.3 ± 3.5	9 ± 4.2	10.3 ± 4.7	+96.6%
	MPresist	J/min	1.6 ± 0.9	2.3 ± 1.1	3.1 ± 1.5	4.3 ± 2.2	5.6 ± 2.7	+248.4%

Observed values are presented as mean ± SD. Protocol A (Complete expiration) adjusted inspiratory-to-expiratory ratios to ensure complete exhalation before the subsequent breath; Protocol B (Fixed I:E) maintained a constant ratio of 1:1.9 throughout. Δ% indicates percentage change from RR 15 min^{-1} to RR 27 min^{-1}. *Abbreviations: VDaw, airway dead space; $PetCO_2$,*

end-tidal carbon dioxide; $\dot{V}CO_2$, carbon dioxide elimination; $VeCO_2$, expiratory CO_2 volume per breath; $ViCO_2$, inspiratory CO_2 volume (rebreathing); Vti, inspiratory tidal volume; Vte, expiratory tidal volume; Ppeak, peak inspiratory pressure; Pplat, plateau pressure; Pmean, mean airway pressure; ΔP, driving pressure; PEEPtotal, total positive end-expiratory pressure; PEEPi, intrinsic PEEP; TI, inspiratory time; TE, expiratory time; InspFlow, inspiratory flow; ExpFlow, expiratory flow; Cstat_calc, static compliance; Rinsp, inspiratory resistance; RCexp, expiratory time constant; MP, mechanical power; MPelastic, elastic component; MPstatic, static component; MPresistive, resistive component.

Table S5. Linear mixed-effects model results with false discovery rate correction.

Outcome	N	R^2 marginal	R^2 conditional	β (RR)	p (RR)	q (RR)	β (RR^2)	p (RR^2)	q (RR^2)	p (Protocol)	q (Protocol)	p (RR* Protocol)	q (RR* Protocol)	p (RR^2* Protocol)	q (RR^2* Protocol)	Rate Ratio (95% CI)	Best-fitting form (AIC)
**A) CO2 Elimination**																	
$PetCO_2$	289	0.096	0.978	-0.019	<0.001	<0.001		-	-	<0.001	<0.001	0.008	0.02	-	-	0.981 (0.979-0.983)	Exp
$\dot{V}CO_2$	289	0.04	0.958	3.851	<0.001	<0.001	-0.198	<0.001	0.002	0.059	0.099	0.539	0.823	0.409	0.784	-	Quad
$VeCO_2$	289	0.058	0.979	-0.150	<0.001	<0.001	-	-	-	0.004	0.01	0.259	0.432	-	-	-	Lin
$ViCO_2$	289	0.126	0.871	0.160	<0.001	<0.001	-0.005	0.031	0.033	0.364	0.454	0.577	0.823	0.47	0.784	-	Quad
$SlopeCO_2$	289	0.004	0.961	0.001	0.856	0.856	-	-	-	0.95	0.95	0.004	0.02	-	-	1.001 (0.995-1.007)	Exp
**B) Dead Space**																	
VDaw	289	0.041	0.945	-0.0153	<0.001	-	-	-	-	0.005	-	<0.001	-	-	-	0.985 (0.981-0.988)	Exp
**C) Volumes**																	
VTi	289	0.005	0.989	-0.476	0.384	0.576	0.130	0.002	0.007	<0.001	<0.001	0.063	0.095	0.01	0.029	-	Quad
VTe	289	0.004	0.983	-0.305	0.617	0.617	0.076	0.109	0.109	<0.001	<0.001	0.016	0.047	0.025	0.037	-	Quad
**D) Pressures**																	
Ppeak	289	0.168	0.909	0.035	<0.001	< 0.001	-	-	-	0.171	0.306	<0.001	< 0.001	-	-	1.036 (1.03-1.041)	Exp
Pplat	279	0.007	0.992	-0.001	0.966	0.966	0.007	0.006	0.009	0.033	0.108	0.696	0.695	0.792	0.79	-	Quad
Pmean	289	0.02	0.993	0.041	0.104	0.131	0.006	0.002	0.005	0.382	0.464	0.259	0.388	0.771	0.79	-	Quad
PEEPi	289	0.267	0.742	-0.039	0.028	0.045	0.004	0.003	0.006	0.561	0.561	0.118	0.246	0.396	0.79	-	Quad
PEEPtotal	289	0.006	0.987	-0.150	<0.001	<0.001	0.013	<0.001	<0.001	0.199	0.306	0.025	0.081	0.346	0.79	-	Quad
ΔP	279	0.015	0.943	0.154	<0.001	<0.001	-0.006	0.046	0.049	0.011	0.073	0.223	0.316	0.652	0.79	-	Quad

Outcome	N	R^2 marginal	R^2 conditional	β (RR)	p (RR)	q (RR)	β (RR^2)	p (RR^2)	q (RR^2)	p (Protocol)	q (Protocol)	p (RR* Protocol)	q (RR* Protocol)	p (RR^2* Protocol)	q (RR^2* Protocol)	Rate Ratio (95% CI)	Best-fitting form (AIC)
**E)Timing & Flows**																	
TI	289	0.906	0.932	-0.098	<0.001	<0.001	0.002	<0.001	<0.001	0.025	0.03	0.006	0.012	0.89	0.89	-	Quad
TE	289	0.944	0.96	-0.131	<0.001	<0.001	0.005	<0.001	<0.001	0.028	0.03	0.01	0.015	0.836	0.89	-	Quad
InspFlow	289	0.489	0.859	0.075	<0.001	< 0.001				0.928	0.928	<0.001	< 0.001	-	-	1.078 (1.07-1.087)	Exp
ExpFlow	289	0.037	0.845	0.011	<0.001	< 0.001				0.928	0.017	0.058	0.058	-	-	1.011 (1.007-1.014)	Exp
**F) Mechanics**																	
Cstat	279	0.009	0.917	-0.01	< 0.001	< 0.001				0.002	0.003	0.02	0.026			0.990 (0.985-0.995)	Exp
Rinsp	279	0.078	0.760	0.041	< 0.001	< 0.001	-	-	-	0.606	0.606	< 0.001	0.001	-	-	1.041 (1.027-1.056)	Exp
RCexp	288	0.038	0.932	-0.011	< 0.001	< 0.001	-	-	-	< 0.001	<0.001	0.004	0.008	-	-	0.989 (0.985-0.993)	Exp
Leak volume	289	0.008	0.85	0.018	0.863	0.863	0.007	0.391	0.397	0.429	0.578	0.688	0.69	0.663	0.667	-	Quad
**G) Mechanical Power**																	
MP	279	0.449	0.950	0.091	< 0.001	< 0.001	-	-	-	0.646	0.721	< 0.001	< 0.001	-	-	1.095 (1.087-1.103)	Exp
MPstatic	289	0.191	0.99	0.220	<0.001	<0.001	0.014	<0.001	<0.001	0.097	0.169	0.03	0.04	0.23	0.47		Quad
MPelastic	279	0.337	0.969	0.201	<0.001	<0.001		-	-	0.275	0.203	< 0.001	0.001	-	-		Lin
MPresistive	279	0.542	0.861	0.168	< 0.001	< 0.001	-	-	-	0.721	0.721	< 0.001	< 0.001	-	-	1.183 (1.164-1.202)	Exp
4·ΔP+RR	279	0.357	0.959	0.162	< 0.001	< 0.001	-0.025	0.049	0.049	0.012	0.061	0.229	0.229	0.655	0.655		Quad

Outcomes were analyzed using mixed-effects models with fixed effects for respiratory rate (RR, centered at 15 min^{-1}), ventilation protocol, and their interaction, plus patient-specific random intercepts and slopes for RR. For each outcome, the optimal functional form (linear, quadratic, or exponential) was selected via Akaike Information Criterion (AIC). Log-transformed (exponential) models were Jacobian-corrected for direct AIC-comparison on the original scale. Model equations: Linear: $Y\_ij = \beta_0 + \beta_1 \cdot RR\_c + \beta_2 \cdot Protocol + \beta_3 \cdot RR\_c \times Protocol + u_{0i} + u_{1i} \cdot RR\_c + \varepsilon_{ij}$; Quadratic: $Y\_ij = \beta_0 + \beta_1 \cdot RR\_c + \beta_2 \cdot RR\_c^2 + \beta_3 \cdot Protocol + \beta_4 \cdot RR\_c \times Protocol + \beta_5 \cdot RR\_c^2 \times Protocol + u_{0i} + u_{1i} \cdot RR\_c + \varepsilon_{ij}$; Exponential: $\log(Y\_ij) = \beta_0 + \beta_1 \cdot RR\_c + \beta_2 \cdot Protocol + \beta_3 \cdot RR\_c \times Protocol + u_{0i} + u_{1i} \cdot RR\_c + \varepsilon_{ij}$. Coefficients (β) represent absolute change per unit for linear/quadratic forms, and change in log(Y) for exponential forms. For the latter, multiplicative Rate Ratios (exp(β)) are provided. Quadratic terms ($\beta\ \_RR^2$) are only reported for AIC-selected quadratic specifications. Marginal/conditional variance explained; β(RR): Linear slope; β(RR^2): Curvature; p/q: Uncorrected/FDR-adjusted

p-values. Rate Ratio: Multiplicative effect of RR (exponential models only). Analysis restricted to RR 15–27 min^{-1} (n=30 patients). Protocol A: Complete Expiration; Protocol B: Fixed I:E. Column definitions: Family, physiologically related outcome group for FDR correction; Type, primary (tested at $\alpha = 0.05$ without correction) or exploratory (FDR-corrected); N, number of observations; R^2 marginal, variance explained by fixed effects; R^2 conditional, variance explained by fixed and random effects; β(RR), linear coefficient for respiratory rate effect (change per 1 min^{-1} increase, on model scale); β(RR^2), quadratic coefficient for quadratic models (positive = accelerating, negative = decelerating pattern); p(RR) and p(RR^2), uncorrected p-values for linear and quadratic terms; q(RR) and q(RR^2), FDR-adjusted q-values; p(Protocol) and q(Protocol), p- and q-values for ventilation protocol main effect; p(RR×Protocol) and q(RR×Protocol), p- and q-values for linear interaction (protocol-specific slopes); p(RR^2×Protocol) and q(RR^2×Protocol), p- and q-values for quadratic interaction (protocol-specific curvature); Rate Ratio (95 % CI), back-transformed multiplicative effect of RR per 1 min^{-1} increase for exponential models only; Best-fitting form (AIC), AIC-selected functional form (Lin = linear, Quad = quadratic, Exp = exponential). Benjamini-Hochberg correction was applied within outcome families (CO_2 elimination, dead space, volumes, pressures, timing/flow, mechanics, mechanical power) to control the false discovery rate at $q < 0.05$. Significant q-values are shown in bold. Analysis restricted to respiratory rates 15–27 min^{-1} (n = 30 patients, 279–289 observations per outcome). *Abbreviations: VDaw, airway dead space; $PetCO_2$, end-tidal carbon dioxide; $\dot{V}CO_2$, carbon dioxide elimination; $VeCO_2$, expiratory CO_2 volume per breath; $ViCO_2$, inspiratory CO_2 volume (rebreathing); Vti, inspiratory tidal volume; Vte, expiratory tidal volume; Ppeak, peak inspiratory pressure; Pplat, plateau pressure; Pmean, mean airway pressure; ΔP, driving pressure; PEEPtotal, total positive end-expiratory pressure; PEEPi, intrinsic PEEP; TI, inspiratory time; TE, expiratory time; InspFlow, inspiratory flow; ExpFlow, expiratory flow; Cstat_calc, static compliance; Rinsp, inspiratory resistance; RCexp, expiratory time constant; MP, mechanical power; MPelastic, elastic component; MPstatic, static component; MPresistive, resistive component.*

Table S6. Functional form comparison for all mixed-effects model outcomes.

Category	Outcome	n	AIC Linear	AIC Quadratic	AIC Exponential	BIC Linear	BIC Quadratic	BIC Exponential
CO_2 Parameters	$PetCO_2$	289	1225.1	1209.9	1200.6	1254.4	1246.5	1229.9
	$SlopeCO_2$	289	684.8	686.7	550.1	714.2	723.4	579.5
	$\dot{V}CO_2$	289	2402.3	2391.0	2398.3	2431.6	2427.6	2427.6
	$VeCO_2$	289	612.9	613.1	625.2	642.2	649.7	654.5
	$ViCO_2$	289	468.4	457.8	458.3	497.7	494.4	487.6
Volumes	VDaw	289	2137.7	2126.6	2109.4	2167.1	2163.2	2138.7
	VTe	289	2292.0	2291.0	2326.7	2321.3	2327.7	2356.0
	VTi	289	2247.6	2242.2	2277.6	2276.9	2278.8	2306.9
Pressures	ΔP	279	631.2	629.3	700.7	660.2	665.6	729.7
	PEEPtotal	289	561.8	515.6	594.2	591.1	552.3	623.5
	PEEPi	289	108.1	86.7	n/a	137.0	122.9	n/a
	Pmean	289	473.8	460.8	493.6	503.1	497.4	523.0
	Ppeak	289	1394.4	1375.4	1322.0	1423.7	1412.1	1351.4
	Pplat	279	576.5	567.8	601.6	605.6	604.1	630.6
Timing	TE	289	−423.4	−578.6	−531.3	−394.1	−541.9	−501.9
	TI	289	−556.8	−609.5	−454.4	−527.5	−572.9	−425.1
Flows	ExspFlow	289	1866.3	1870.1	1536.2	1895.6	1906.7	1565.6
	InspFlow	289	2262.1	2258.6	1934.5	2291.4	2295.2	1963.8
Mechanics	Cstat	279	2087.2	2086.1	1876.8	2116.3	2122.4	1905.8
	RCexp	288	−863.6	−860.4	−939.5	−834.3	−823.8	−910.2
	Rinsp	279	1221.3	1221.5	1152.8	1250.4	1257.8	1181.9
	Leak volume	289	1081.5	1080.8	n/a	1110.2	1116.7	n/a
Mechanical Power	MP	279	1461.4	1427.1	1206.2	1490.4	1463.4	1235.2
	MPelastic	279	195.3	198.8	226.6	224.3	235.1	255.7
	MPresistive	279	1376.6	1352.9	908.6	1405.7	1389.2	937.7
	MPstatic	289	579.6	529.3	553.3	608.9	566.0	582.6
	4·ΔP+RR	279	1404.7	1402.8	1457.1	1433.8	1439.1	1486.2

For each outcome, three functional forms were compared (linear, quadratic, and exponential, with log-transformed outcome), all retaining identical random-effect structures (random intercept and slope per patient) and protocol moderation. Akaike Information Criterion (AIC) and Bayesian Information Criterion (BIC) for log-transformed outcomes were Jacobian-corrected to allow direct comparison on the original outcome scale. AIC was prioritized over BIC as the latter’s stricter penalty tends to under-fit non-linear responses at moderate sample sizes.

Figure S10. Residual diagnostics for the mechanical power and carbon dioxide elimination ($\dot{V}CO_2$) models with ventilatory ratio interaction.

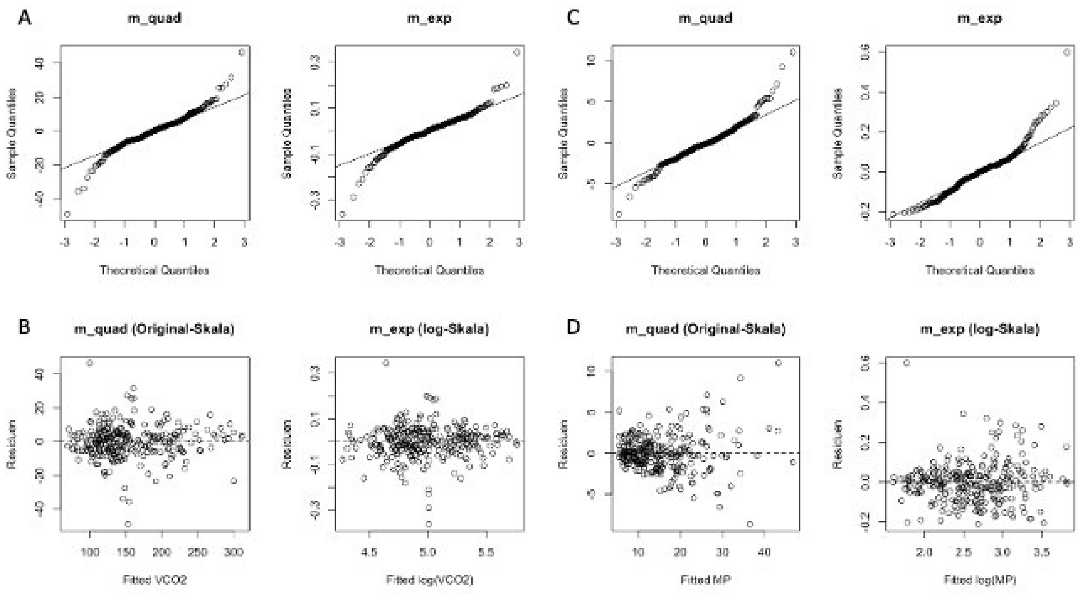

Residuals from quadratic (m_quad) and log-linear (m_exp) specifications for CO_2 elimination (VCO_2; A, B) and mechanical power (MP; C, D). **(A)** VCO_2, Q-Q: residuals follow the theoretical normal line across the central distribution on both scales, with mildly heavy tails at both extremes. **(B)** VCO_2, residuals vs. fitted: residual variance is approximately constant across the fitted range on both scales. **(C)** MP, Q-Q: residuals deviate from the theoretical line at both tails on the original scale and follow it closely on the log scale, with one positive outlier remaining. **(D)** MP, residuals vs. fitted: residual variance increases approximately fivefold across the fitted range on the original scale and stabilizes after log-transformation.

Table S7. Linear mixed-effects model for mechanical power and carbon dioxide elimination ($\dot{V}CO_2$) with ventilatory ratio interaction.

Parameter	log(MP)		$\dot{V}CO_2$		4·ΔP+RR	
	β (95% CI)	p	β (95% CI)	p	β (95% CI)	p
**Observations (n)**	269		279		269	
Fixed effects						
Intercept	2.6988 (2.5916 to 2.8060)	<0.001	157.447 (143.317 to 171.577)	<0.001	43.643 (40.462 to 46.824)	<0.001
RR (centred)	0.0914 (0.0839 to 0.0988)	<0.001	1.524 (0.816 to 2.233)	<0.001	1.563 (1.329 to 1.797)	<0.001
RR^2 (centred)	—	—	-0.172 (-0.257 to -0.088)	<0.001	-0.019 (-0.037 to -0.002)	0.031
Protocol B (Fixed I:E 1:1.9)	-0.1398 (-0.1674 to -0.1123)	<0.001	3.129 (0.594 to 5.665)	0.016	1.304 (0.410 to 2.199)	0.004
VR (centred)	0.9696 (0.4973 to 1.4420)	<0.001	169.350 (106.214 to 232.487)	<0.001	16.031 (2.045 to 30.017)	0.026
RR × Protocol B	-0.0250 (-0.0316 to -0.0184)	<0.001	1.480 (0.875 to 2.086)	<0.001	-0.173 (-0.299 to -0.047)	0.007
RR × VR	-0.0083 (-0.0382 to 0.0215)	0.573	3.066 (0.169 to 5.964)	0.039	-0.244 (-1.255 to 0.766)	0.635
RR^2 × VR	—	—	-0.489 (-0.871 to -0.107)	0.012	0.043 (-0.035 to 0.122)	0.279
Random effects						
Subject (random intercept), variance	0.0742		1340.10		63.735	
Subject (random RR slope), variance	0.0002 (corr = 0.28)		2.144 (corr = 0.79)		0.034 (corr = 0.19)	
Residual, variance	0.0129		113.37		4.732	
Model fit						
Marginal R^2	0.638		0.483		0.357	
Conditional R^2	0.948		0.960		0.959	
ICC (adjusted)	0.857		0.923		0.936	

Linear mixed-effects models with subject-specific random intercepts and RR slopes were fitted separately for log-transformed mechanical power [log(MP)] and CO_2 elimination ($\dot{V}CO_2$). Fixed effects included RR (centered at 15 min^{-1}), protocol (A: complete expiration [reference] vs. B: fixed I:E 1:1.9), baseline ventilatory ratio (VR), and RR × protocol and RR × VR interactions. The $\dot{V}CO_2$ model additionally included quadratic RR terms based on AIC-guided model selection among linear, quadratic and exponential specifications (Table S6). Models were fitted by maximum likelihood using bobyqa ($\dot{V}CO_2$) and Nelder–Mead (log[MP]) optimizers. Coefficients for log(MP) are reported on the log scale; 95% CIs are Wald-type with Satterthwaite degrees of freedom. Marginal and conditional R^2 and adjusted ICC were calculated according to Nakagawa & Schielzeth. The log(MP) model included 269 observations from 28 subjects, and the $\dot{V}CO_2$ model 279 observations from 29 subjects.

Table S8. Linear mixed model results for marginal changes in ventilatory parameters.

A: Fixed Effects						
**Outcome**	**Term**	**Estimate**	**SE**	**95% CI**	**t value**	**p value**
Δ$\dot{V}CO_2$ (mL/min)	Intercept	23.854	4.864	[14.18, 33.53]	4.91	<0.001
	RR	-0.670	0.214	[-1.10, -0.24]	-3.13	0.002
ΔMP (J/min)	Intercept	0.086	0.487	[-0.88, 1.05]	0.18	0.861
	RR	0.115	0.020	[0.08, 0.15]	5.78	<0.001

B: Random Effects		
**Outcome**	**Patient (Intercept)**	**Residual**
Δ$\dot{V}CO_2$ (mL/min)	3.839	7.143
ΔMP (J/min)	1.061	0.652

Linear mixed models were fitted with respiratory rate as fixed effect and random intercepts for individual patients (n = 29, 103 observations). Section A shows fixed effects estimates with standard errors and 95% confidence intervals. Section B shows random effects variance components, where Patient (Intercept) represents between-patient variability and Residual represents within-patient variability across respiratory rate steps. Δ$\dot{V}CO_2$ = change in CO_2 elimination between consecutive respiratory rate steps; ΔMP = change in mechanical power between consecutive respiratory rate steps.

Sensitivity analyses

Safety parameter analyses confirmed robustness for Ppeak (observed increase: 53.8% Complete Expiration, 16.9% Fixed I:E) and $PetCO_2$ (observed decrease: 20.6% Complete Expiration, 18.0% Fixed I:E). For PEEPi in complete expiration protocol, the low baseline values (0.4 ± 0.24 mbar) resulted in high sensitivity to imputation assumptions, with a tipping point at δ=2 mbar; however, absolute changes remained clinically negligible across all scenarios (<0.6 mbar at RR27).

Signal stability

After Hochberg correction, no systematic drift was detected for any variable at any frequency level (29/30 tests $p_{adj} > 0.05$). CVs remained below 5% across all conditions (Table S9). These findings confirm that two minutes of equilibration were sufficient to reach steady state and that volumetric capnography provided stable measurements even at higher respiratory rates.

Table S9. Summary of sensitivity analyses for missing not at random (MNAR) assumptions.

Outcome	Protocol	N at RR27	Dropout	Observed Δ%	Worst-Case Δ%	Stat. Tipping Point	Clin. Tipping Point	Robust?
**Ppeak**	1	24 (80%)	6	+53.8%	+51.6%	δ=62	δ=0 (50%)	Yes
	2	28 (93%)	2	+16.9%	+19.8%	δ=0 (significant)	δ=10 (21%)	Yes
**PEEPi**	1	24 (80%)	6	+7.5%	+2.9%	δ=2	δ=2 (57%)	Sensitive
	2	28 (93%)	2	+138%	+131%	None	δ=0 (130%)	Yes
**$PetCO_2$**	1	24 (80%)	6	-20.6%	-8.7%	δ=0 (significant)	None	Yes
	2	28 (93%)	2	-18%	-11.8%	δ=0 (significant)	None	Yes

Sensitivity analyses were conducted for safety parameters (Ppeak, PEEPi, $PetCO_2$) across both ventilation protocols. N at RR27 indicates the number of patients with available data at respiratory rate 27 min^{-1}. Observed Δ% represents the percentage change from RR15 to RR27 based on observed data only. Worst-Case Δ% shows results when missing values were imputed at 150% of baseline. Stat. Tipping Point denotes the delta value (δ) at which the respiratory rate effect became statistically significant ($p<0.05$); "None" indicates the effect remained non-significant up to δ=100. Clin. Tipping Point indicates the delta value at which percentage change exceeded the predefined clinical relevance threshold of 20%. Results were considered robust when conclusions remained stable across all tested MNAR scenarios, and sensitive when small changes in assumptions substantially altered results.

Figure S11A-G. Observed group means across the full respiratory rate range (available-case analysis)

A) CO_2 parameters

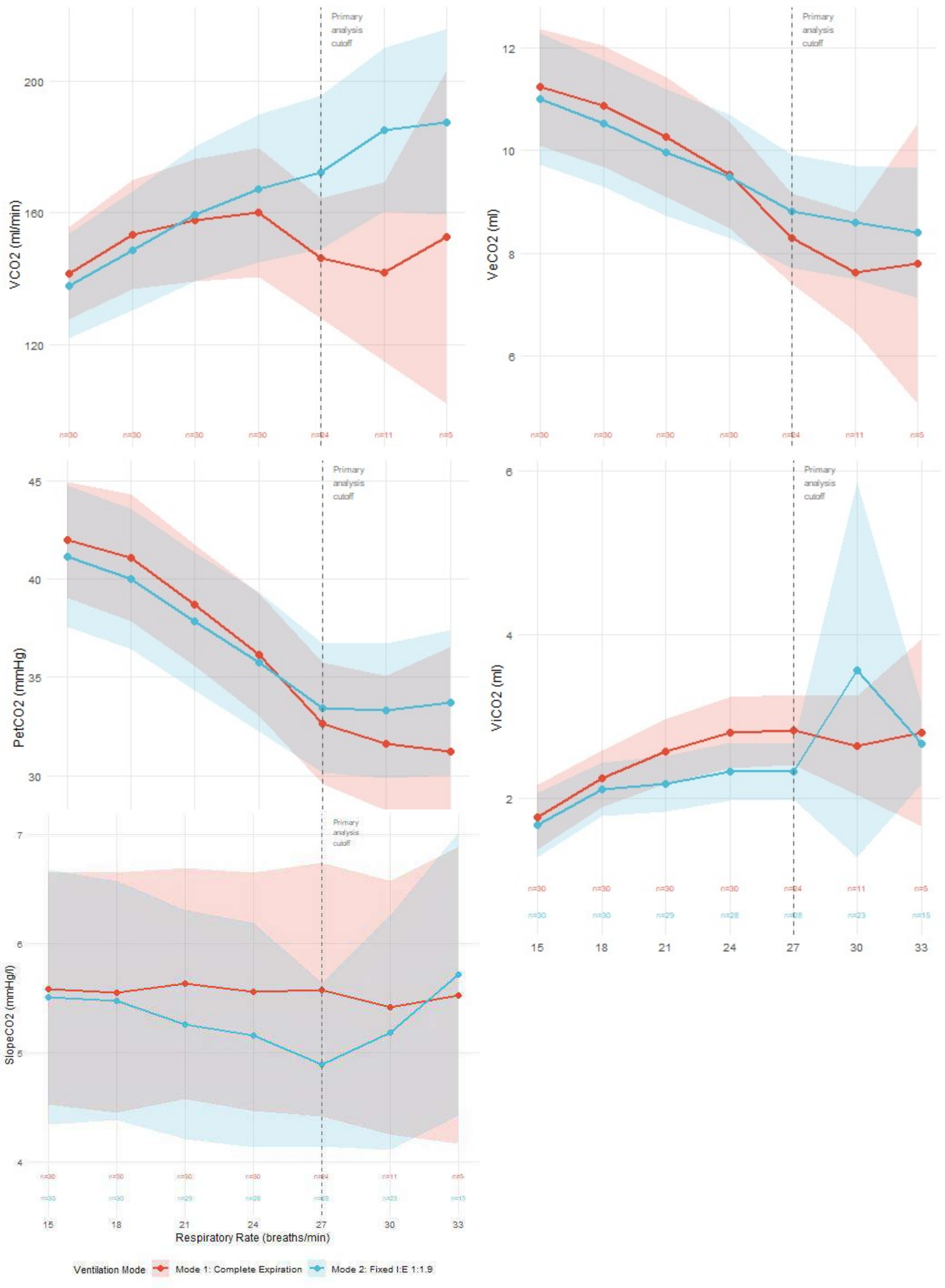

B) Dead space

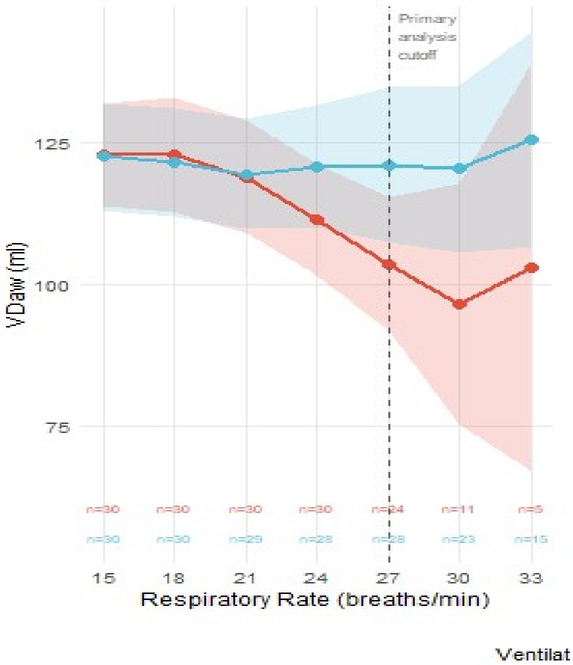

C) Tidal volumes

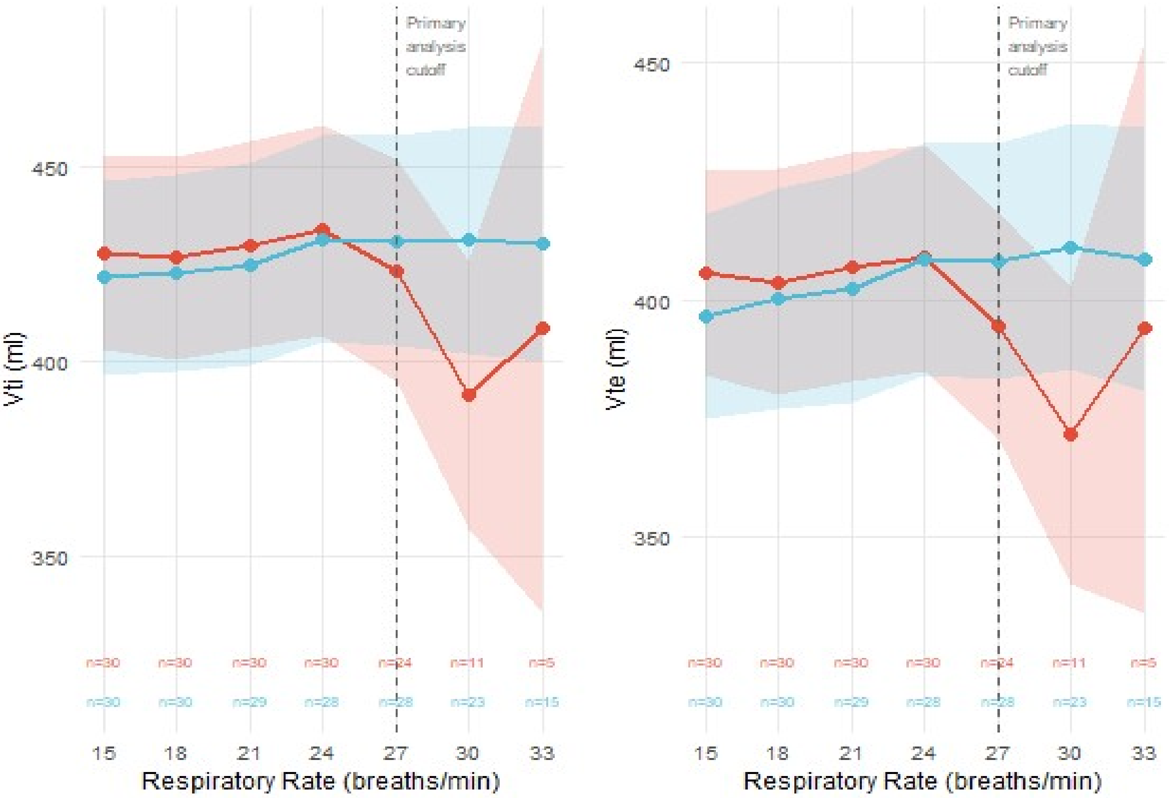

D) Airway pressures

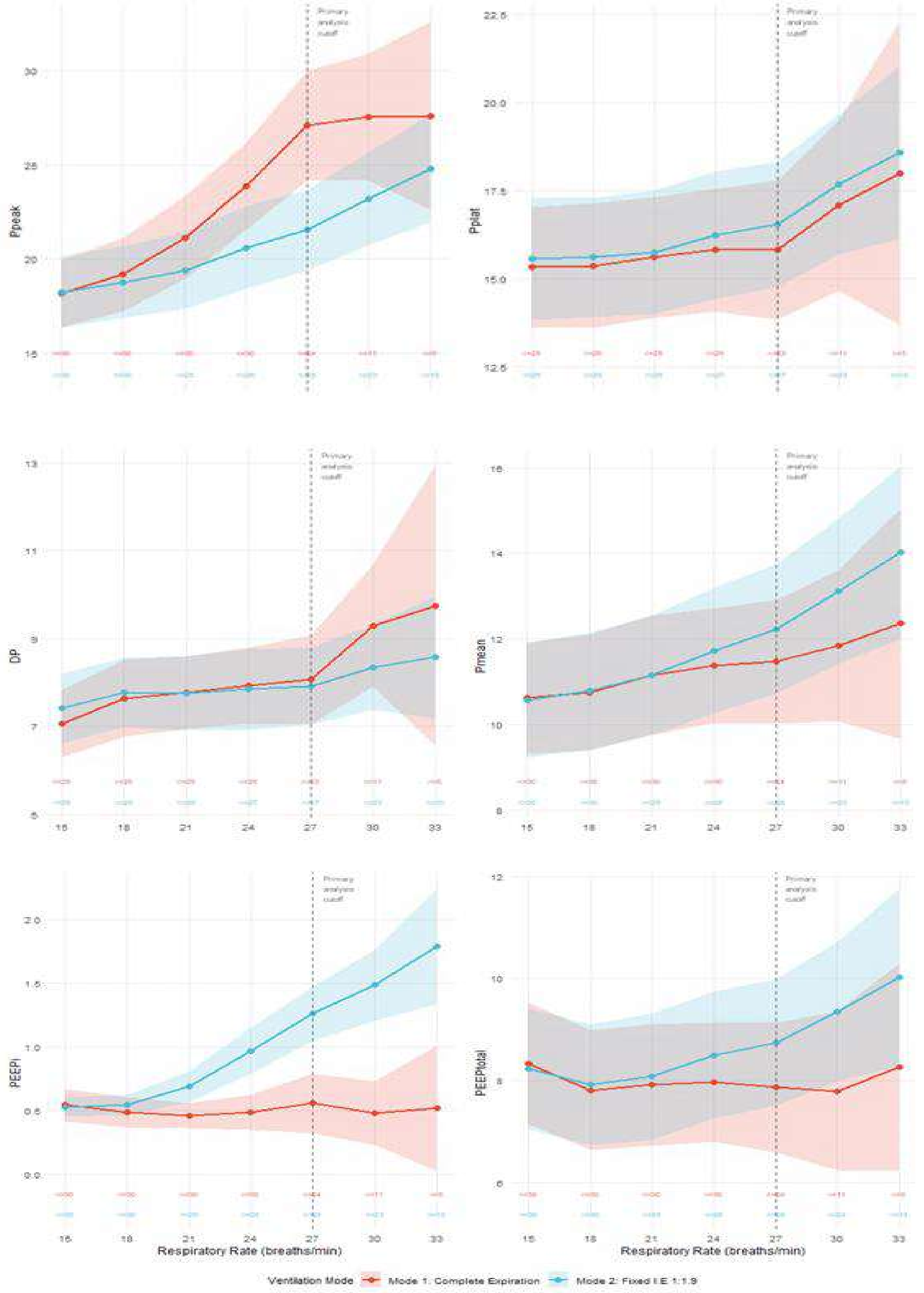

E) Timing and Flow parameters

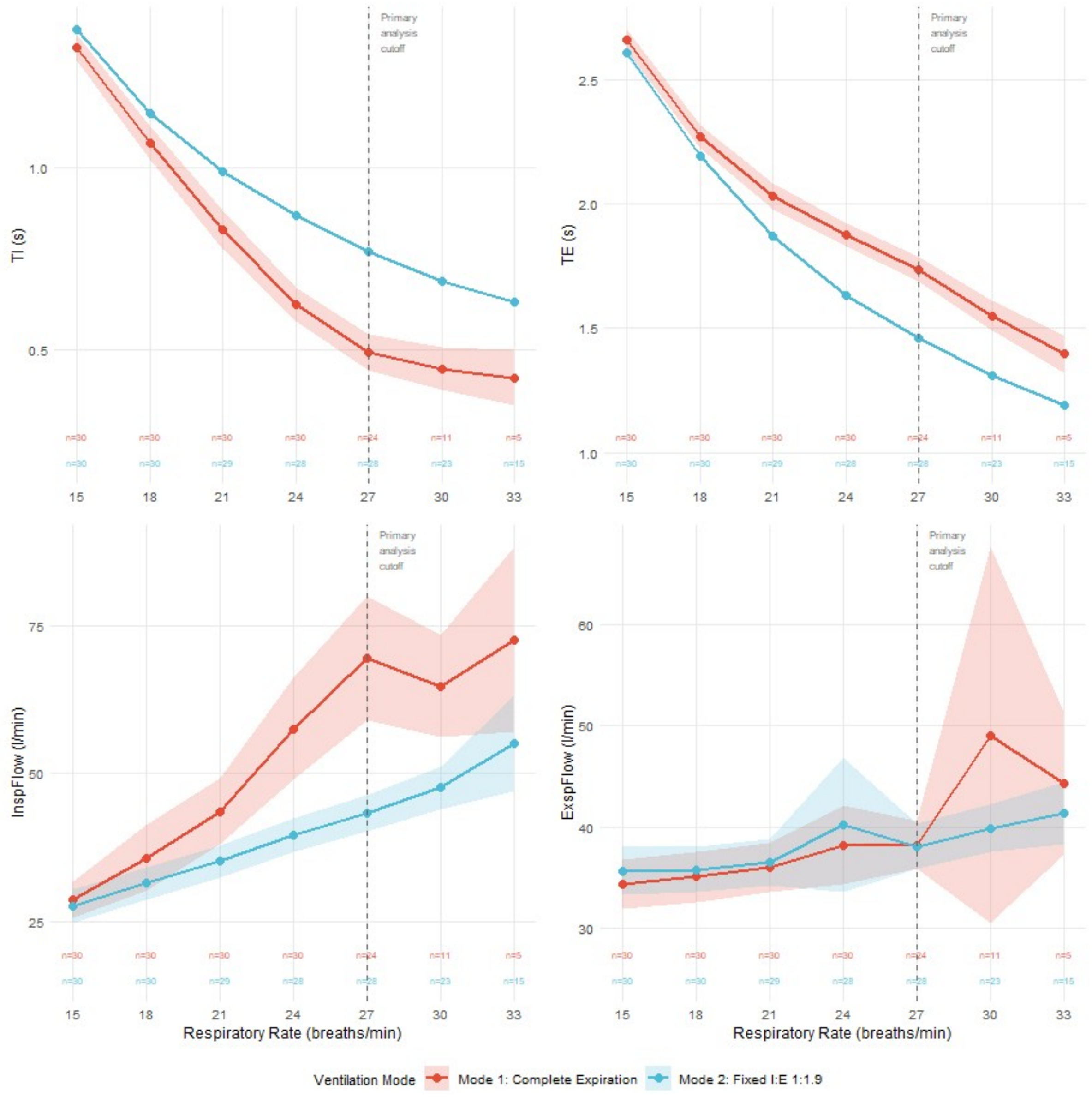

F) Respiratory system mechanics

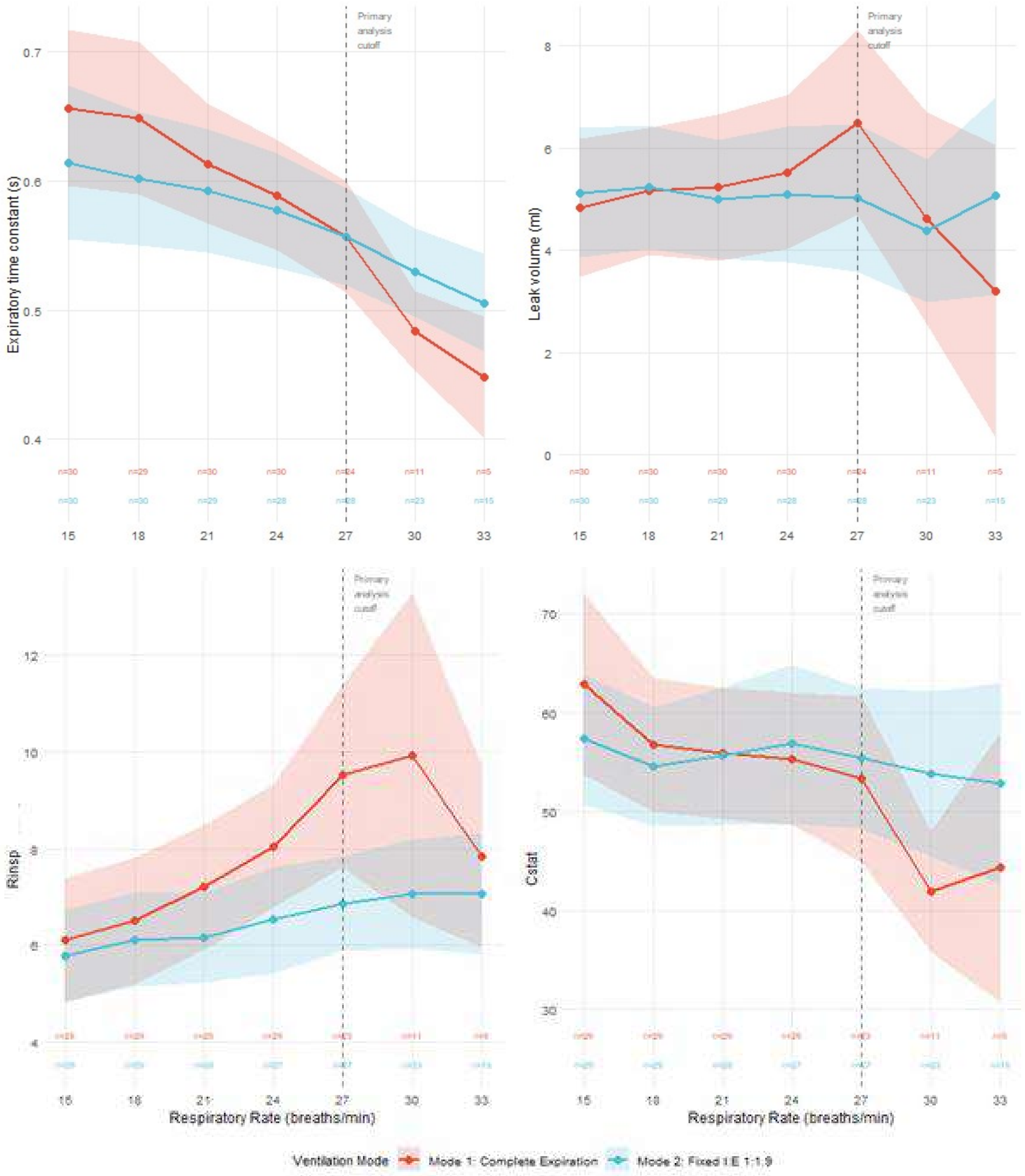

G) Mechanical power components

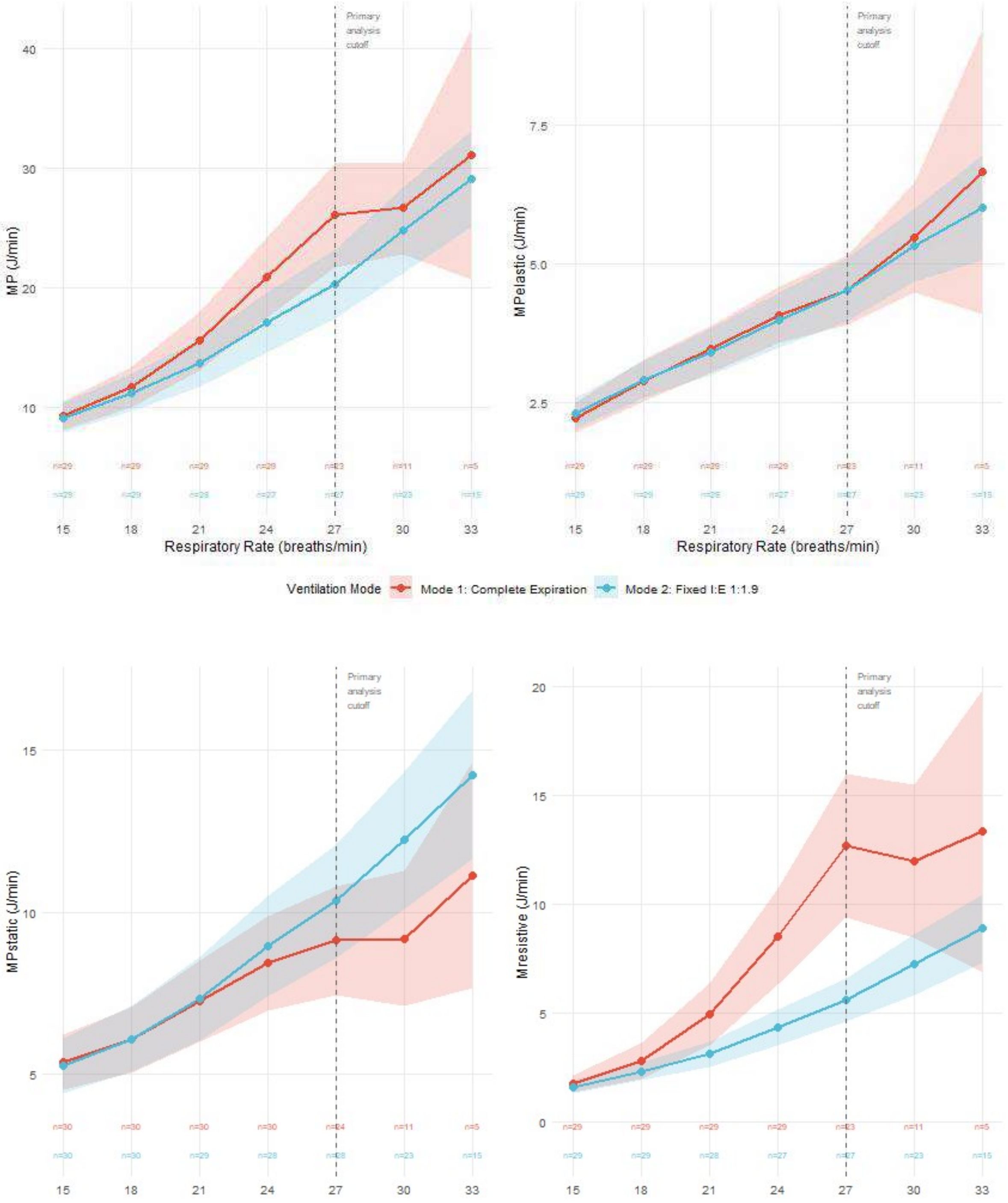

In contrast to Figure 2, which presents model-based estimated marginal means restricted to the pre-specified primary analysis range (RR 15–27 min^{-1}), Figures S11A-G display observed group means across the full respiratory rate range (15–33 min^{-1}) without modelling, allowing transparent visualization of data behaviour beyond the analysis range and protocol-specific dropout patterns. Group means with 95% confidence intervals are shown (red: complete expiration; blue: fixed I:E 1:1.9). The dashed vertical line indicates the primary analysis cutoff

(RR ≤27 min^{-1}); values beyond this threshold represent a selected subpopulation of patients who tolerated higher respiratory rates and should be interpreted with caution. Sample sizes at each respiratory rate level are displayed below the x-axis. Complete expiration maintained full retention through RR 24 min^{-1}, followed by steep attrition, whereas fixed I:E showed earlier but more gradual dropout, retaining more patients at RR 30–33 min^{-1}. Panels: (A) CO_2 parameters; (B) Dead space; (C) Tidal volumes; (D) Airway pressures; (E) Timing and flow parameters; (F) Respiratory system mechanics; (G) Mechanical power components All airway pressures (Ppeak, Pplat, Pmean, PEEP, PEEPi, ΔP) are given in mbar; derived metrics use the corresponding mbar-based units (Rinsp in $mbar \cdot s \cdot L^{-1}$, Cstat in $mL \cdot mbar^{-1}$).

Figure S12. Treatment Response Heterogeneity to Minute Ventilation Augmentation.

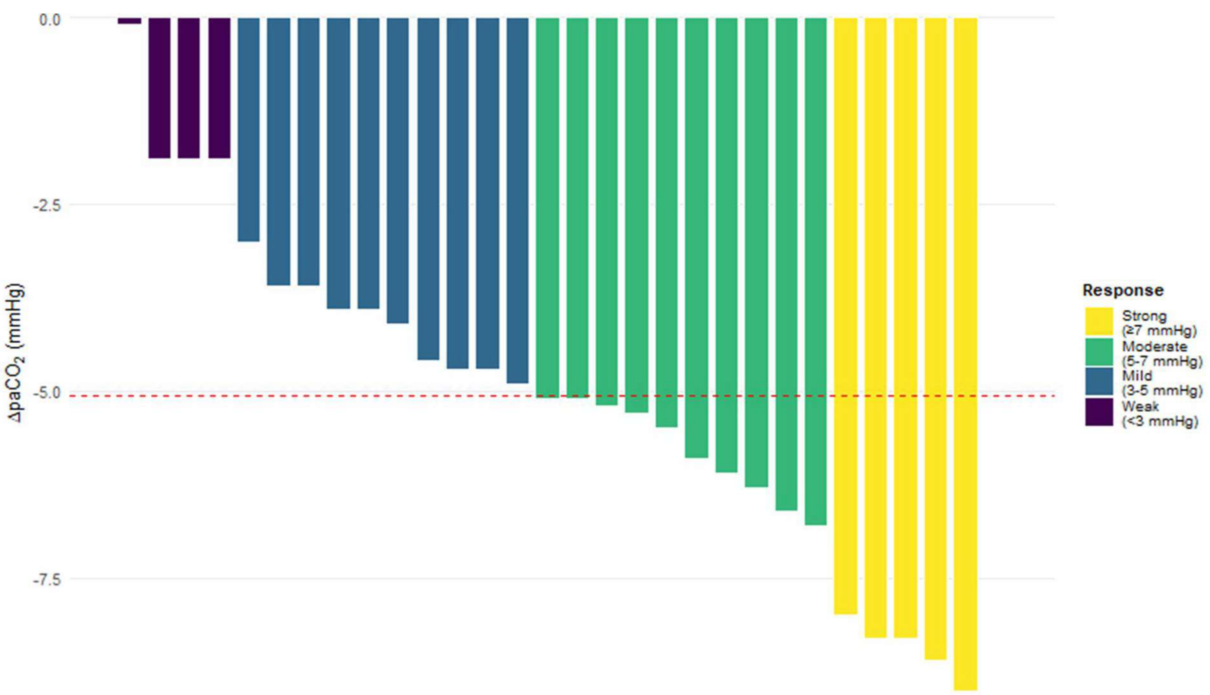

Individual patient responses to respiratory rate-driven minute ventilation increase from 100 to 150 mL/kg IBW. The x-axis represents individual patients (n = 29), ranked by their $PaCO_2$ response from largest reduction (left) to smallest reduction (right); this ordering is applied identically across all panels to visualise inter-variable relationships. ΔPaCO2 ranged from −8.5 to −1.2 mmHg, indicating substantial interindividual variability. Responses were categorized as follows: strong (≥7 mmHg reduction), moderate (5–7 mmHg), mild (3–5 mmHg), and weak (<3 mmHg). The dashed red line represents the mean $\Delta PaCO_2$.

Table S10. Signal stability of volumetric capnography parameters across respiratory rate levels.

Variable	Metric	RR 15	RR 18	RR 21	RR 24	RR 27
**VDaw (ml)**	CV (%), median (IQR)	2.44 (1.63–3.56)	2.55 (1.63–3.70)	2.74 (1.94–3.80)	2.65 (2.13–4.30)	2.90 (2.16–4.44)
	Drift (%/breath), median (IQR)	−0.031 (−0.199–0.262)	0.133 (−0.157–0.410)	0.027 (−0.288–0.416)	−0.112 (−0.558–0.221)	−0.064 (−0.482–0.208)
	p (Hochberg-adjusted)	0.968	0.968	0.968	0.968	0.968
**SlopeCO$_2$ (%CO$_2$/L)**	CV (%), median (IQR)	1.96 (1.42–3.66)	2.37 (1.48–3.54)	2.00 [1.39–3.70]	1.74 (1.15–3.60)	2.13 (1.45–3.71)
	Drift (%/breath), median (IQR)	0.085 (−0.407–0.556)	−0.062 (−0.538–0.421)	−0.137 (−0.509–0.338)	0.059 (−0.156–0.345)	−0.100 (−0.578–0.195)
	p (Hochberg-adjusted)	0.968	0.968	0.968	0.968	0.968
**$\dot{V}CO_2$ (ml/min)**	CV (%), median (IQR)	0.00 (0.00–1.66)	0.00 (0.00–1.63)	0.00 (0.00–1.93)	0.00 (0.00–1.35)	0.00 (0.00–1.57)
	Drift (%/breath), median (IQR)	0.000 (0.000–0.000)	0.000 (0.000–0.000)	0.000 (−0.293–0.000)	0.000 (0.000–0.000)	0.000 (0.000–0.000)
	p (Hochberg-adjusted)	0.968	0.968	0.016	0.968	0.968

Coefficient of variation (CV) and normalized drift (slope) are reported as median [IQR] across all 30 patients, pooled across both ventilation protocols. CV quantifies breath-by-breath variability within each two-minute measurement window. Drift represents the normalized slope of consecutive measurements (% change per breath), with values near zero indicating absence of systematic signal trends. Drift was tested against zero using one-sample Wilcoxon signed-rank tests with Hochberg correction for 15 comparisons. After correction, 14 of 15 tests were non-significant (p~adj~ > 0.05); the single nominally significant result ($\dot{V}CO_2$ at RR 21, p~adj~ = 0.016) showed a median drift of zero with a marginally asymmetric IQR, which was not considered physiologically meaningful. All CVs remained below 5%. $VeCO_2$ and $ViCO_2$ were excluded from this analysis because their integer-valued output (ml) at the low per-breath volumes in this cohort precluded meaningful variability assessment.

References

1. Grasselli G, Calfee CS, Camporota L, Poole D, Amato MBP, Antonelli M, u. a. ESICM guidelines on acute respiratory distress syndrome: definition, phenotyping and respiratory support strategies. Intensive Care Med. Juli 2023;49(7):727–59. doi:10.1007/s00134-023-07050-7

2. Qadir N, Sahetya S, Munshi L, Summers C, Abrams D, Beitler J, u. a. An Update on Management of Adult Patients with Acute Respiratory Distress Syndrome: An Official American Thoracic Society Clinical Practice Guideline. Am J Respir Crit Care Med. 1. Januar 2024;209(1):24–36. doi:10.1164/rccm.202311-2011ST

3. Hohmann F, Fichtner F, Becher T, Schaedler D, Putensen C, Muders T, u. a. Clinical Guideline for Treating Acute Respiratory Insufficiency with Invasive Ventilation and Extracorporeal Membrane Oxygenation: Updated Evidence-Based Recommendations for Choosing Modes and Setting Parameters of Mechanical Ventilation. Respiration. 11. Dezember 2025;1–17. doi:10.1159/000549732

4. Boussarsar M, Thierry G, Jaber S, Roudot-Thoraval F, Lemaire F, Brochard L. Relationship between ventilatory settings and barotrauma in the acute respiratory distress syndrome. Intensive Care Med. April 2002;28(4):406–13. doi:10.1007/s00134-001-1178-1

5. Wolff G, Brunner J, Weibel W, Bowes C, Muchenberger R, Bertschmann W. Anatomical and series dead space volume: concept and measurement in clinical practice. Appl Cardiopulm Pathophysiol ACP. 1989;2:299–307.

6. Costa ELV, Slutsky AS, Brochard LJ, Brower R, Serpa-Neto A, Cavalcanti AB, u. a. Ventilatory Variables and Mechanical Power in Patients with Acute Respiratory Distress Syndrome. Am J Respir Crit Care Med. 1. August 2021;204(3):303–11. doi:10.1164/rccm.202009-3467OC
